# Evaluation of bond durability of different self-adhesive bioactive restorative systems to dentin

**DOI:** 10.1038/s41598-024-81351-9

**Published:** 2025-01-29

**Authors:** Fawkia M. Samy, Naglaa R. El-Kholany, Hamdi H. Hamama

**Affiliations:** Conservative Dentistry Department, Faculty of Dentistry, Mansoura University, Mansoura, Egypt

**Keywords:** Alkasite, Self-adhesive hybrid composite, Self-adhesive restorative materials, Microshear bond strength, Micromorphological analysis, Dental caries, Restorative dentistry, Bonded restorations

## Abstract

This study aimed to compare the bonding efficacy three bioactive self-adhesive restorative systems to dentin. A total of 80 permanent human molars were utilized in this study. The occlusal enamel was removed to exposed mid-coronal dentin; 40 molars were used for microshear bond strength testing, while the remaining molars were used for micromorphological analysis of restoration/dentin interface. Accordingly, 4 groups were assigned according to the used restorative materials; (G1) self-adhesive hybrid composite (surefil one), (G2) Alkasite-based material (Cention forte) without pretreatment primer, (G3) Alkasite-based material (Cention forte) with pretreatment primer (Cention primer), and (G4) resin-modified glass ionomer (fuji II LC). Then each group was divided into 2 sub-group according to testing time (n = 5); immediate (after 24 h) and delayed (after 6 months of storage in artificial saliva). Microshear bond strength testing employed a universal testing machine to quantify the force required for material fracture at the interface, followed by failure mode analysis. Interfacial micromorphology was assessed using scanning electron microscopy (SEM). In µSBS, the outcome of Two-way ANOVA showed that, there is a statistically significant difference in “type of the restorative material” and “storage time” (p˂0.05. The output of Tukey post-hoc test revealed highest µSBS values were recorded in both immediate and delayed was recorded for Cention Forte with it’s pretreatment primer (p < 0.05). Whereas Surefil one & Cention Forte (without primer) showed the lowest µSBS results among its immediate and delayed groups (p < 0.05). Regarding the micromorphological patterns of restoration/dentin interface using SEM, there was a difference among the tested groups. This study revealed that using of primers prior to application of alkasite-based restorative material is highly recommended as this techniques seems to be the most effective in obtaining superior bond strength with dentin. Accordingly, this outcome of this study highlighting the importance of using primer in enhancing bonding to dentin, which might slightly countered the initial manufacturer’s recommendations and categorization of this type of restorations as a self-adhesive.

## Introduction

Utilizing bioactive ‘ion-releasing’ restorative materials might help in solving many challenges of bonding to caries-affected dentin (CAD). Furthermore, these materials can achieve durable bonding between restorative materials and CAD tooth substrate which is essential for the long-term clinical success for dental restorations^1^. Traditionally, bonded restorations is mediated by dental adhesives; however, self-adhesive restorative materials represent a significant advancement for direct restorative materials^1^. These materials offer simple application technique and potentially overcoming challenges associated with complex adhesive protocols^2^. Furthermore they reduce chair-side time and decrease cumulative iatrogenic errors by eliminating pre-treatment adhesive step^3,4^.

The current philosophies of minimal invasive dentistry highly recommend using bioactive ‘ion-releasing’ restorative. This refers to the capacity of the material to promote biomineralization through the strategic release of essential ions^5^. Bioactive materials offer significant advantages in restorative dentistry, they enhance the longevity of restorations, stimulate dentin repair mechanisms, and improve interfacial adhesion. Consequently, the incidence of recurrent caries and marginal leakage is significantly reduced^6^.

Glass ionomer cements (GICs) has a distinguished position among dental restorative materials due to their inherent bioactivity^7^^.^ This pioneering characteristic facilitates a direct interaction with hard tooth structures through a self-adhesion mechanism^8^. This material was considered as the gold standard for self-adhesive direct restorations^9^. Conventional glass ionomer cements (GIC) were developed on the basis of silicon and polycarboxylic cements in the late 1960s^5^. Resin-modifies GI restorations was introduced as an attempt to overcome drawbacks of conventional GICs; relatively long setting time, inferior mechanical properties, and moisture sensitivity^5^. This modified GIC incorporating 2-hydroxyethylmethacrylate (HEMA) to orginal formula to the enhance color stability and bonding to tooth substrate^9^. Both conventional and resin-modified glass-ionomer cements (GICs) inherently bond to tooth structure via shallow hybridization (micromechanical interlocking) as well as, ionic bonding of carboxyl groups of polyalkenoic acid with calcium ions of hydroxyapatites^10^.

Composite restorations are highly demanded in daily dental practice mainly due to their esthetical and mechanical properties. Composite resins have been used in dentistry for approximately 50 years^11^, and exhibited some shortcomes; some of these materials allowing plaque formation as well as bacterial proliferation which consequently increases the prevelance of recurrent caries^12^.

These innovative milestone of introducing novel hybrid materials that are referred to as ‘self-adhesive’, ‘bulk-fill’, or ‘ion-releasing’ restorative material, represent a significant advancement in concept of minimally invasive restorative dentistry. These novel restorative materials eliminate the need for separate adhesives, reducing the chance of contamination from blood or saliva. Additionally, they address potential issues associated with adhesives, such as post-operative sensitivity. The bulk-fill concept is another step towards simplification and in same time most of these materials exhibit an ion-releasing ability. Several studies^13,14^ reported the benefits of ion-releaseproperties on remineralization and the prevention of dental caries.

A recent development in dental restorative materials is the introduction of self-adhesive resinous composites with claimed fluoride-releasing and “bulk-fill” properties. These materials, categorized as “bioactive” or “smart”, possess a distinct chemical composition compared to the established glass ionomer cement (GIC) family. Initial investigations suggest they have the potential to exceed the performance of GICs^9^.

Alkasite-based material is a new category of restorative materials represents an attempt for manufacturing a potentially bioactive restorative materials^5^. Alkasite-based material is describes as a hybrid tooth-colored restorative material. It is manufactured to release calcium, fluoride, and hydroxyl ions, which show a potent anti-cariogenic properties. This new-class of material combines favorable characteristics of glass ionomer cements (GICs) and resin-based composites. It offers dual-cure functionality, allowing for bulk placement with or without an adhesive layer^15^. The similarity of the alkasite-based material to a regular resin-based composite is given by the monomer matrix and some of it’s inorganic fillers^16^. It contain alkaline fillers (SiO2-CaO-CaF2-Na2O glass; 24.6 wt%) that release acid-neutralizing ions to prevent demineralization of the teeth^16^. These fillers^17^ are the origin of the name ‘alkasite’ given by the manufacturer.^5^

Additionally, self-adhesive hybrid composite was introduced in 2019 categorized as an advanced self-adhesive restorative (ASAR). It was introduced as promising restorations^5^ that combined ion-releasing^18^ and self-adhesive capabilities of glass ionomer cements in resinous nature. This is achieved via unique MOPOS (Modified Polyacid System) monomer that initiates adhesion and enhances material strength, alongside BADEP (bifunctional acrylates), which facilitates cross-linking and covalent bond formation. The self-adhesive hybrid composite material was discovered to have similar mechanical properties to clinically established posterior restorative materials, including flexural strength, fatigue strength, flexural modus, and fracture toughness, as well as comparable wear resistance to resin base composites^17^. The manufacturer claims that the capacity to release calcium, aluminum, and fluorine ions is another characteristic of a self-adhesive composite^5^. According to the manufacturer’s tests, it exhibits a long-term release of fluoride that is similar to the long-term releases of fluoride by GIC and RM-GIC (even after 450 days)^5^.

Multiple investigations demonstrated that alkasite-based restorative materials exhibit significantly superior compressive, tensile, and shear bond strengths compared to glass-ionomer cements (GICs)^19–22^. Furthermore, the manufacturers of these materials often position them as comparable to amalgams in terms of compressive strength and durability, while offering ion-releasing properties similar to GICs^21^. Additionally, the increased translucency of alkasite materials is touted as an aesthetic advantage over GICs^23^. However, it’s important to note a relative lack of studies directly comparing the performance of alkasite restorations with other contemporary esthetic restorative materials.

Some studies investigated a novel self-adhesive hybrid composite material for use in posterior dental restorations. The material exhibited mechanical properties comparable to clinically established restorative materials, including flexural strength, fatigue strength, flexural modulus, and fracture toughness. Furthermore, it demonstrated wear resistance comparable to resin-based composites^17^. While the self-adhesive properties to enamel and dentin resembled those of glass-ionomer cements and modern adhesives^24,25^. Further research is necessary to definitively determine the bond strength of this new bulk-fill restorative material (Supplementary Table S1).

The rapid proliferation of novel dental materials presents a significant challenge for clinicians seeking to optimize patient outcomes^26^. However, a critical gap exists in the current evidence base regarding the bond strength of bioactive restorative materials. To address this knowledge deficit and guide clinical decision-making, a meticulously designed laboratory study was warranted to evaluate the performance of these contemporary materials (alkasite & Self-adhesive bulk-fill hybrid composite) compared to RMGICs.

A laboratory study has shown no difference in the dentin shear bond strength between self-adhesive bulk-fill composite and these of RM(GIC)^1^. However, other studies^9,10^ revealed different clinical and laboratory performances between these self-adhesive restorative materials. A laboratory study^9^ has shown difference in shear bond strength and interfacial surface formation between self-adhesive bulk-fill composite, alkasite compared to HV-GICs.

A key question this study aimed to answer was whether these self-adhesive restorative materials will be sufficiently efficient to bond to dentin? A critical evaluation of self-adhering restorative materials necessitates assessment of both immediate and long-term bonding efficacy to dentin. Scanning electron microscopy (SEM) is commonly employed for morphological analysis of adhesive-dentin interfaces. Comprehensive studies have explored the correlation between bonding performance and interfacial characteristics including hybrid layer formation, integrity, and thickness, as well as resin tag morphology^27,28^, so scanning electron microscopy (SEM) had been used to gain valuable insights into the effectiveness of the adhesive interface and ensure optimal bonding for long-lasting dental restorations^29^.

This study aimed to compare the bond durability, microshear bond strength, and interfacial micromorphology of various self-adhesive bioactive restorative systems bonded to dentin. The null hypothesis of this study was that there would be no significant difference in these properties between the different materials tested.

## Materials and methods

The full description of materials used in the current study is illustrated in Table 1.

**Table 1:** Materials used in this study.

Materials	Specification	Manufacturer	Composition	Application	Code batch no. (lot)
Surefil one	Self-adhesive bulk-fill resinous restorative material with ionic release after polymerization No bonding agent required regardless of cavity	Dentsply Sirona, Konstanz, Germany	Aluminum-phosphor-strontium-sodium-fluoro-silicate glass, highly dispersed silicon dioxide, ytterbium fluoride, iron oxide pigments, titanium dioxide pigments ,polycarboxylic acid, acrylic acid, bifunctional acrylate, water, self-cure initiator, camphorquinone, stabilizer	1. Activate the capsule 2. Mix it with an amalgamator for 10s 3. Injected directly by capsule applier 4. Light cure for 20s with an output of 1200 mW/cm2 → Self-cure for 6 min (prior to further specimen processing)	(‘SU-O’) 2,201,000,713
Cention forte	“Alkasite” bulk-fill resinous restorative material with ionic release after polymerization under acidic challenge Bonding agent (Cention primer) not required for retentive cavities but required for a non-retentive cavities	Ivoclar Vivadent; Schaan, Liechtenstein	Barium aluminum silicate glass, ytterbium trifluoride, pre-polymerized filler, calcium barium aluminum fluorosilicate glass, and calcium fluoro-silicate glass,UDMA, tricyclodecan-dimethanol dimethacrylate, tetramethyl-xylylene diurethane dimethacrylate, polyethylene glycol, 400 dimethacrylate, and Ivocerin	1. Actively scrub and agitate the primer for 10s 2. Dry with compressed air until a glossy thin immobile layer remains 3. Activate the capsule 4. Mix it with an amalgamator 5. Extrude directly by capsule applier → Self-cure, optionally speed up the process by light cure for 15s	(‘CNF’) ZL08SV
Fuji II LC	Resin-modified glass-ionomer Self-adhesive resinous restorative material with ionic release No bonding agent required, regardless of cavity	GC; Tokyo, Japan	Fluoro-alumino-silicate glass, Polybasic carboxylic acid, UDMA, HEMA, Water,Inatiator	1.Apply Dentin Conditioner for 20 s 2. Activate the capsule 3. Mix it with an amalgamator for 10s 4. Injected directly by capsule applier 5. Light cure for layers of max. 1.8-mm thickness for 20s with an output of 1200 mW/cm2 .	(‘FJI’) 2,302,132
Cention primer	A two-component self etching and self-curing primer	Ivoclar Vivadent, Schaan, Liechtenstein	Liquid: bisphenol A glycerolate dimethacrylate, 2-hydroxyethyl methacrylate, methacrylated phosphoric acid, 1,10-decandiol dimethacrylate, methacrylate modified polyacrylic acid, 2-dimethylaminoethyl methacrylate, ethanol, camphorquinone	1.Applied to the dentin surface with a single-use applicator. 2.Coating and scrubbing for 10 s 3.The primer was dispersed with compressed air until a thin and shiny film had formed	(‘p’) Z031Z2
Dentin conditioner	Polyacrylic acid-etch	GC; Tokyo, Japan	(GC; 20% polyacrylic acid, 3% aluminum chloride, distilled water)	1.Spplied for 20 s to the dentin surface of the specimen. 2.Rinse thoroughly with water and dry gently	(‘DC’) 5,040,518

### Teeth selection

Eighty freshly extracted intact, non-carious, and unrestored human permanent molars were collected from the outpatient clinic of the Faculty of Dentistry, Mansoura University. The reason for extraction was due to periodontal diseases. Calculus and soft-tissue deposits were removed with a hand scalar (Nordent, Ivory #2e3, USA), The molars were cleaned using a rubber cup (prophy rubber polishing cup, China), and fine pumice water slurry (PSP, Dylan Rd, Belvedere, England) and then stored in 0.5% chloramine T solution at 4 ℃ (Faculty of Pharmacy, Mansoura University, Egypt) for 24 h after extraction. The storage procedures in distilled water till use performed following the current International and institutional infection control guidelines. The protocol of collection and storage of extracted teeth was approved by Faculty Ethical Committee under Ref # A09030123.

### Sample size calculation

Sample size calculation was based on mean bond strengths between different groups of treatment of the dentin surface retrived from previous research (Sadeghyar et al., 2022)^30^. Using G power program version 3.1.9.7 to calculate sample size based on effect size of 0.35, using 2-tailed test, α error = 0.05 and power = 90.0%, the total calculated sample size will be 120 total.

### Specimen preparation

The roots of each specimen were embedded in self-cure acrylic resin (Acrostone, Heliopolis, Cairo, Egypt) up to 2 mm below the cement-enamel junction (CEJ) using cylindrical polyvinyl chloride (PVC) tubes(2 × 2.7 cm) as molds. For each tooth, a standardized mid-coronal dentin exposure was created by removing a predetermined thickness of occlusal enamel and superficial dentin. The mid-coronal dentin location was determined using preoperative radiographic measurements and marked on the tooth’s external surface.

The cutting procedure was made perpendicular to the longitudinal axis of each tooth using a low-speed automated diamond saw (PICO 155 precision saw, Pace technologies, Tucson, AZ, USA) with presence of water coolant (Diacut Water-based Cutting Fluid Pace technologies, Tucson, AZ, USA) at a concentration of 1: 33, lubricant: water during the cutting procedure. The exposed dentin surfaces were finished by using 600-grit silicon carbide paper (Microcut, Buehler, Lake Bluff, IL, USA) in a rotational motion for 30 s under running water to create a standardized smear layer.

### Grouping specimens

**Fig. 1:**
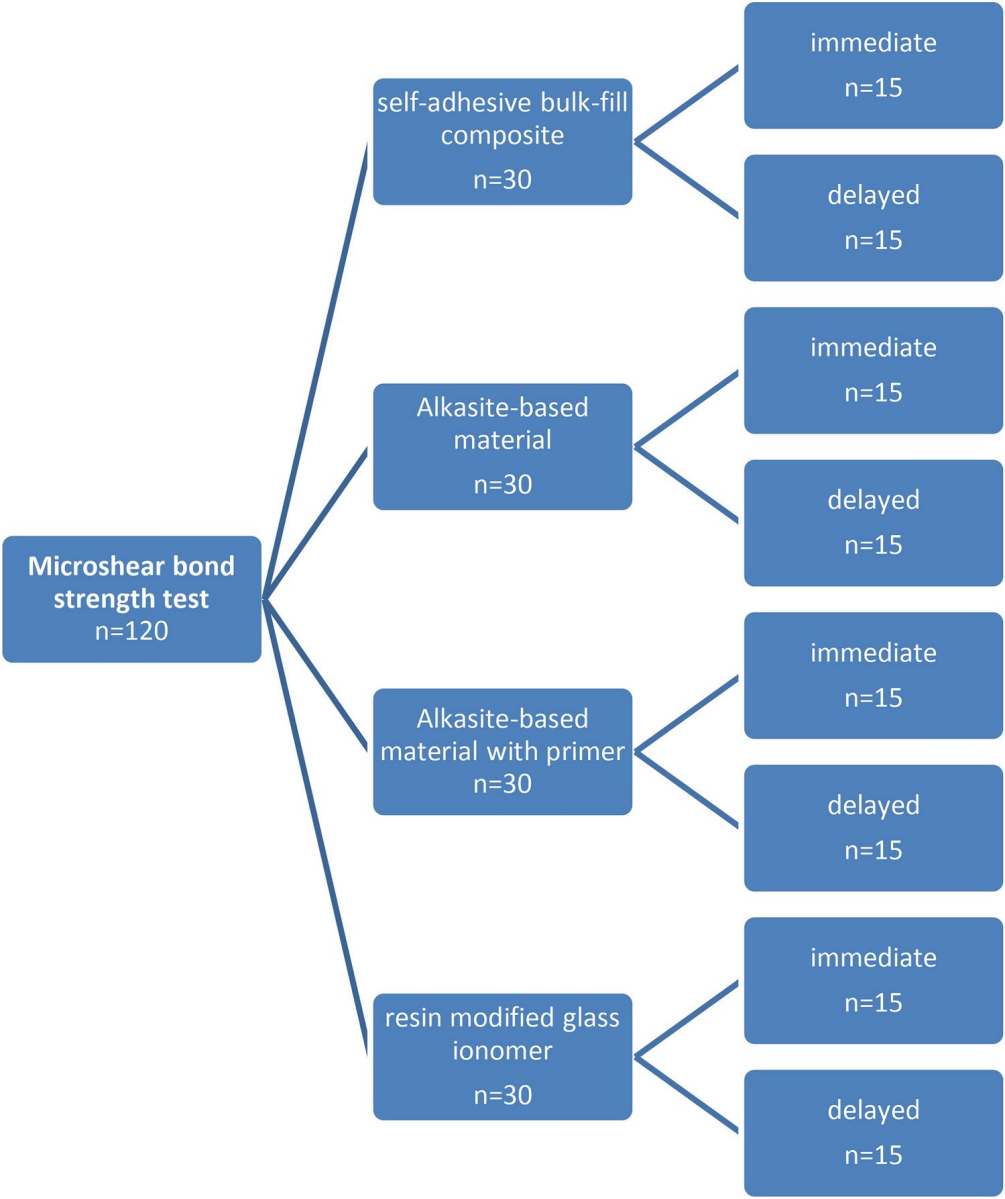
Diagram showing the study design for micro shear bond strength test.

Total forty molar(M) teeth used in this study, For µSBS test, were randomly divided into 4 groups in order to the type of studied restorative material and pretreatment agent; (Each group had 10 molars every molar had 3 cylinders of restorative material (n = 30)): group A; surefil one (self-adhesive bulk-fill composite group, Dentsply Sirona, USA]), group B; Cention fort without primer (Alkasite-based material group, Ivoclar Vivadent, Schaan, Liechtenstein), Cention fort with primer (Alkasite-based material group) and Fuji II LC (resin-modified glass ionomer GC; Tokyo, Japan, control group). Each group (10 M) was divided into 2 sub-groups (5M) according to storage time; immediate specimens (n = 15) were evaluated after 24h, while the delayed groups (n = 15) were evaluated after 6-month storage in artificial saliva (Faculty of pharmacy, Mansoura University, Egypt) to determined bond durability of the materials (Fig. 1). The other forty molars (n = 40) were tested for micromorphological analysis under SEM of restoration/dentin interface and assigned into four groups (n = 10) according to the tested restorative material. Each group (n = 10) was divided into 2 sub-groups according to storage time; immediate specimens (n = 5) were evaluated after 24h, while the delayed groups (n = 5) were evaluated after 6-month storage in artificial saliva (Fig. 3). The materials employed in the current study are demonstrated in Table 1**.**

### Measurement of microshear bond strength

Addition silicon (polyvinylsiloxane) impression material in super light consistency (Ghenesyl, Super Light Body, Lascod, Florence, Italy) was acted as mold for the three self-adhesive restorative materials on each spaceman through creating a cylinder of rubber base with 2 mm thickness at the edges, 1 mm thickness on the prepared dentin surface and 2.5 cm diameter by using a cylindrical plastic measuring cap base (EL Badr plastic co., Alexanderia, Egypt)^31^. The inner surface of rubber base cylinder had a depression accommodating each on the dentin surface which assisted in the fabrication of restorative material micro-tubes, three marks were done at the depression of the rubber base cylinders, then these marks were drilled with 1 mm diameter cylindrical end-cutting diamond bur ISO #150 (Osung Dental, USA) with medium grit at 45,000 rpm/min the alignment of the bur during the drilling procedure was adjusted by using dental surveyor that had handpiece holder (MARATHON Surveyor 103 complete) with micromotor, each resulted hole was (1 mm diameter and 1mm height) and the space between each hole and the adjacent was 2 mm. The diameter of each hole and the space between each hole and the adjacent were measured using a digital caliper.

For the self-curing Alkasite-based material (CNF) group with primer, a Cention Primer was applied to the dentin surface with a single-use applicator. After coating and scrubbing for 10 s, the primer was dispersed with compressed air until a thin and shiny film had formed. For Resin-modified glass ionomer (Fuji II LC) group a dentin Conditioner was applied for 20 s to the dentin surface of the specimen, rinse thoroughly with water and dry gently.

After completion of the pretreatment steps, The previously fabricated rubber base molds were realigned over their specimens and three restorative materials (‘SU-O’), (‘CNF’), (‘FJI’) were condensed into the holes by using a rounded blunt end (1mm in diameter) of periodontal probe and finally adapted by small ball burnisher, Glass slide was used to compress the material to extrude extra material and decrease surface voids then coated with a translucent polyester strip [0.12 mm thickness] before light curing according to manufacturing instructions using LED light-curing unit (Elipar TM Deep Cure-S LED Curing Light) with a power of 1200 W/cm and a wavelength range of 350–520 nm.

**Fig. 2:**
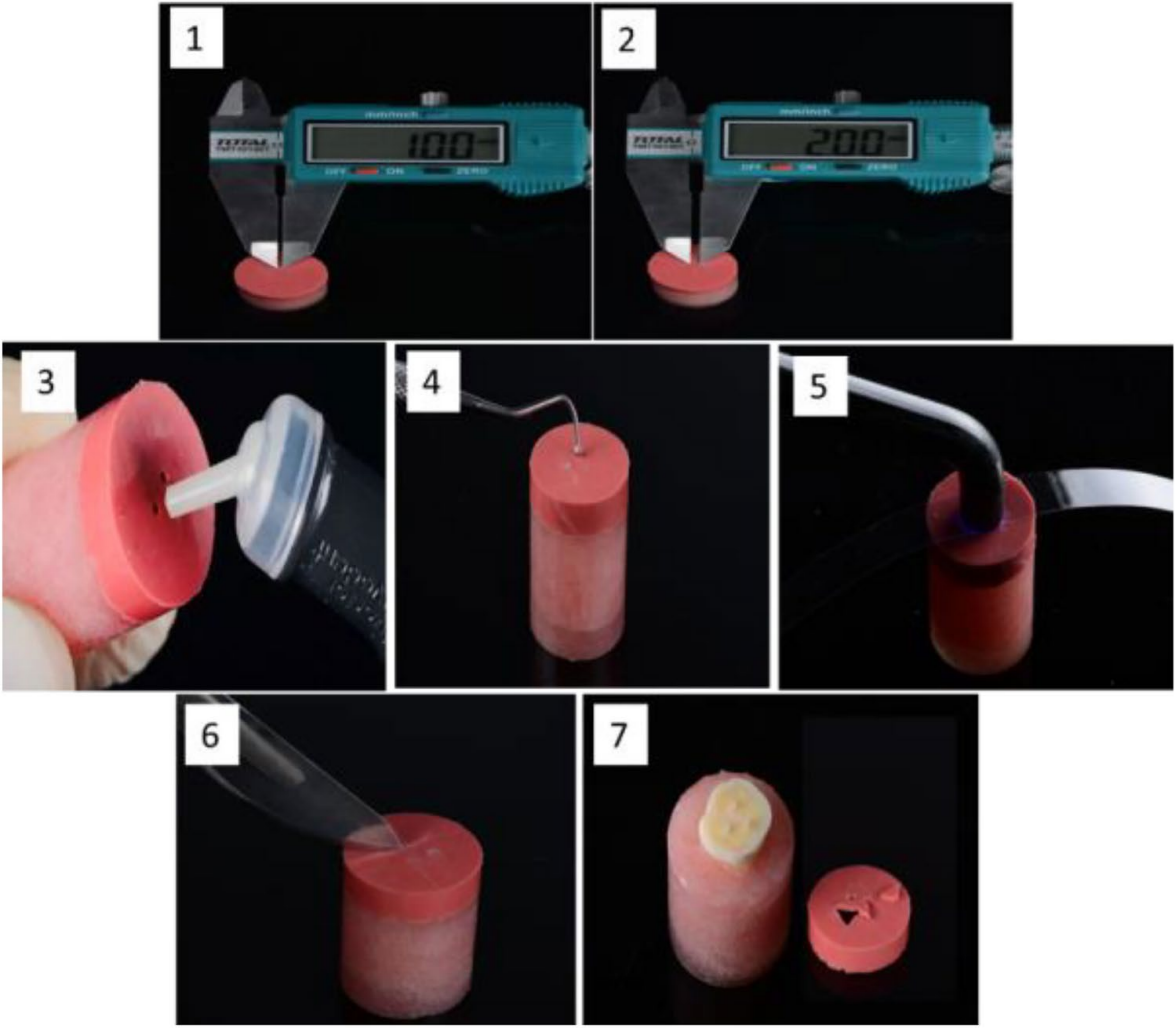
Application of three restorative materials (‘SU-O’), (‘CNF’), (‘FJI’) and removal of rubber mold. (**1**) & (**2**) the diameter of created hole (1mm) and the space between each hole and the adjacent (2mm) were measured using a digital caliper. (**3**) application of restorative material.(**4**) restorative materials (‘SU-O’), (‘CNF’), (‘FJI’) were condensed into the holes by using a rounded blunt end (1mm in diameter) of periodontal probe and finally adapted by small ball burnisher. (**5**) covering the holes with a translucent polyester strip [0.12 mm thickness] before light curing according to manufacturing instructions using LED light-curing unit (Elipar TM Deep Cure-S LED Curing Light) with a power of 1200 W/cm and a wavelength range of 350–520 nm. (**6**) Cutting the addition silicon mold After the restorative materials completely set by scalpel to prevent pretest failure of the recently created.

All restorative materials were prepared, and applied on the dentin surface according to the manufacturers’ instructions (Table 1). After the material set, the mold was removed by a scalpel, as shown in Fig. 2, it was removed after 24 h to verify the setting of the materials, especially since some used materials are chemically cured (Alkasite-based materials) while others are dual cured (RMGICs), and any excess material was gently removed from around the base of the material cylinder with a scalpel.

Then three tubes were obtained each cylinder was 1 mm in length and 1 mm in diameter, all material cylinders’ diameter (cross sectional surface area) was measured using a digital caliper.

The immediate specimens (n = 15) were stored in water at 37 ℃ for 24 h and then were evaluated. While the delayed specimens (n = 15) were evaluated after 6-months of storage in artificial saliva, that was prepared at faculty of pharmacy at Mansoura university, at 37 ℃ in an incubator to determined bond durability of the materials. Artificial saliva was changed weekly for 6 months of storage.

#### Mounting the specimen to universal testing machine

The specimens of each group were tested for microshear bond strength test using a universal testing machine (Model 3345, Instron, USA). Each specimen was mounted to the lower fixed compartment of universal testing device with tightening screws, A loop was prepared from thin orthodontic wire (Dentaurum, Turnstr, Ispringen, Germany) [0.2 mm in diameter] was hocked around material cylinder/dentin interface and attached to the upper moving arm of the testing machine. Load was applied to each cylinder specimen at a crosshead speed of 1 mm/min until fracture occurred (The load was increased at a rate of 1 mm/min until the specimen failed), and the machine’s software was used to analyse the stress strain curve. The micro shear bond strength value in megapascals (MPs) was calculated as the load at failure (Newton) divided by the cross-sectional area of the material cylinder (π r^2^) in mm2**.** The separation sites of all tested specimens were examined by a stereomicroscope (SZX10; Olympus, Japan) at × 40 magnification to determine the mode of failure that classified as; “- Type CF-D: Cohesive failure within the dentin—Type AF: Adhesive failure at the interface between the material and dentin—Type MF: Mixed failure (adhesive and cohesive failure within the material)—Type CF-M: Cohesive failure within the restorative material –PTF: failure during artificial aging period” ,all data were collected and subjected to statistical analysis. Representative samples from each failure mode were gold-coated and examined using a scanning electron microscope (SEM, JSM-6510LV SEM, JEOL Ltd., Tokyo, Japan) at a magnification of approximately 50 × or 55x.

### Micromorphological analysis of restoration/tooth interface

#### Specimen grouping

**Fig. 3:**
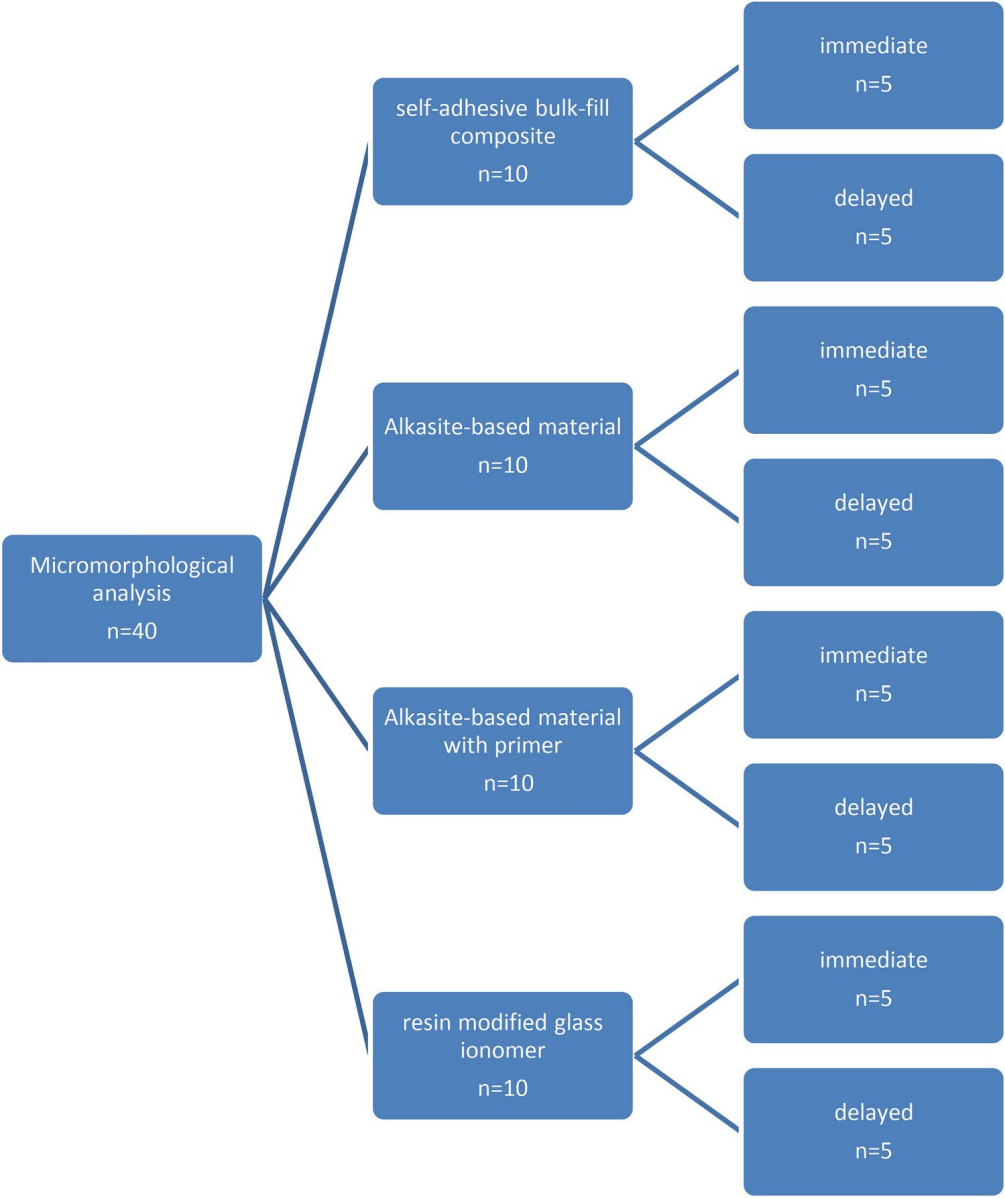
Diagram showing study design for micromorphological pattern.

Additional 5 molars from each subgroup were prepared with a total no. of 40 teeth from all groups were obtained, according to the previously mentioned study design (for microshear bond strength test). The immediate subgroup (I) was stored in distilled water at 37 ℃ for 24 h was evaluated, while the delayed subgroup (D) was kept in artificial saliva for 6 months. (Fig. 3).

#### Specimen preparation

The enamel surface and superficial dentin was removed to expose the mid-dentin using a low-speed automated diamond saw (PICO 155 precision saw, Pace technologies, Tucson, AZ, USA) with presence of water coolant. The cut surface was subjected to silicon carbide paper with grit 600 in order to obtain a standardized smear layer (this as for microshear bond strength test although). Then the assigned self-adhesive restorative materials were applied on the prepared surface; all restorative materials were prepared, and applied on the dentin surface according to the manufacturers’ instructions; with 2mm height using tofflemire matrix system, which was adjusted to provide a 2mm height of restorative material as measured by a periodontal probe, and glass slide was used to compress the material to extrude extra material and decrease surface voids. The metal band was removed after the material had fully cured, then the specimens were kept at room temperature in distilled water for 24 h before the sectioning.

The molars were vertically sectioned bucco-lingually, after 24 h from application of the restorative materials, into two roughly equal halves along their long axes in a perpendicular direction to the restoration dentin interface and then the teeth were horizontally sectioned at level of CEJ to be separated from The acrylic resin using a diamond disc at low speed (PICO 155 precision saw, Pace technologies, 34th St. Tucson, AZ 85,713 USA). While employing water coolant (Diacut Water-based Cutting Fluid, Pace technologies, Tucson, AZ, USA) at a concentration of 1:33, lubricant: water during the cutting procedure.

Although Resin-dentin slabs, obtained from each half were subsequently polished with silicon carbide papers (Microcut, Buehler, Lake Bluff, IL, USA) of varying grits (600, 1000, 1200, 2000, and 4000-grits, respectively), The final polish was achieved with diamond pastes of decreasing sizes [6, 4, and 1 m, respectively] (Metadi, Buehler, Lake Bluff. IL, USA) by using a piece of polishing cloth. The samples were put in Ultrasonic bath (CD-4820 ultrasonic cleaner, Codyson, Shenzhen, China) for 10 min in order to eliminate any debris.

For acid–base challenge, The specimens were stored at room temperature in a saline solution for 10 min before being exposed to a 10% orthophosphoric acid solution for 5 s and then to a 5% sodium hypochlorite for 5 min, This technique demineralized any dentin that was not infiltrated by resin so that the dentin could be dehydrated. The samples were dried and kept dry for 24h hydration during the teeth gold plating. Specimens were gold sputtered (SPI Module—Sputter Carbon / Gold Coater, EDEN instruments, Japan) and observed in secondary electron detection mode under scanning electron microscopy (SEM) (JSM6510LV, JEOL, Japan) at an accelerating voltage of 30 kV an working distance of 10–15 mm, Several SEM images were taken at different magnifications. Initially, a low-magnification SEM image was captured. Among all the images, the most informative and clearest was the one taken at a magnification of × 1000. This methodology followed the published paper by Hamama et al.^32^.

### Statistical analysis

#### Statistical methods

Data were tabulated and coded using (Microsoft Excel 2016). Data analysis was performed using Statistical package for social science (SPSS 22, SPSS Inc, Chicago, Illinois, USA).

#### Sample size calculation

Using G power program version 3.1.9.7 to calculate sample size based on effect size of 0.35, using 2-tailed test , α error = 0.05 and power = 90.0%, the total calculated sample size was be 120 specimens.

#### Data expression

The normality of the quantitative data distribution was statistically assessed using the Shapiro–Wilk test, as the sample size per group was less than 50. The p-value was greater than 0.05 (p > 0.05), indicating that the data were normally distributed.

#### Data comparison

In order to compare data, two-way ANOVA (analysis of variance) was used for evaluating numerical (parametric) data involving more than two different group, then post-hoc Tukey tests were conducted.

## Results

### Microshear bond strength (μSBS) test

**Fig. 4:**
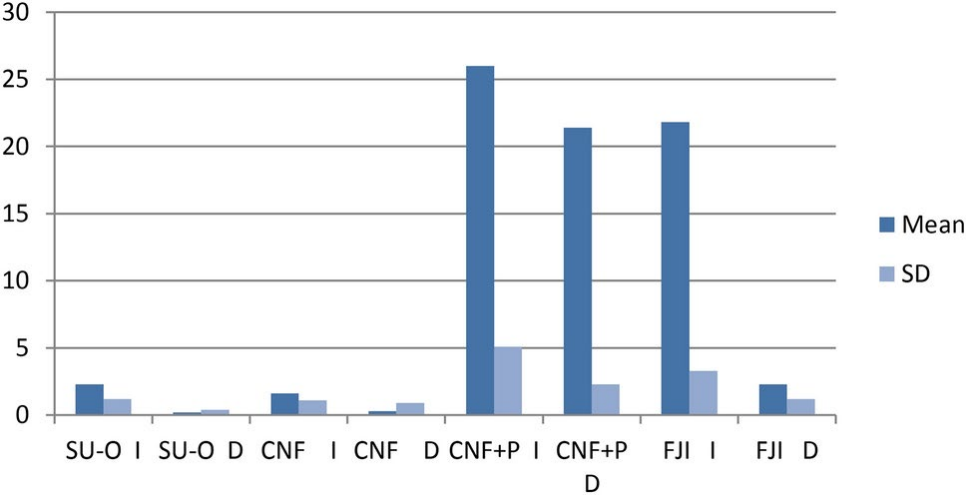
Mean µSBS values ± standard deviation for all test groups.

For all groups, the distribution of data was statistically checked by Shapiro–Wilk test showed that the μSBS data followed a normal distribution pattern (p > 0.05). The outcomes of the two-way ANOVA test revealed that the μSBS (Microshear bond strength) mean values are affected significantly by “type of the restorative material” and “storage time” (p˂0.05) Also, the interaction between the two factors (type of the restorative material* storage time) was significant (p˂0.05) (Table 2). The µSBS mean values in Mega-Pascal and standard deviations for all groups with Tukey’s HSD post-hoc multiple comparisons between groups are presented in (Table 2) and (Fig. 4).

**Table 2:** Means (MPa) and Standard deviation (SD) values for different groups and multiple comparisons between means.

Group			Mean (N) ± SD (MPa)
Material	Time	n	
SU-O	I	15	2.1587 ± 1.24608^C^
	D	15	0.2060 ± 0.37127^C^
CNF	I	15	1.5540 ± 1.09871^C^
	D	15	0.3333 ± 0.86874^C^
CNF + P	I	15	26.0360 ± 5.14593^A^
	D	15	21.3773 ± 2.77064^B^
FJI	I	15	21.7860 ± 3.30991^B^
	D	15	2.2880 ± 1.17500^C^

Means are arranged from A to C with A are the highest values and C are the lowest values. Means with the same superscripted letters have no significant differences (Tukey HSD; p˃0.05).

*SU-O* Surefil One, *CNF* Cention Forte without Cention primer, *CNF + P * Cention Forte with Cention primer, *FJI* Fuji II LC, *I* immediate, *D* Delayed.

For immediate (un-stored) groups, Tukey’s HSD post-hoc multiple comparison test results showed that the CNF + P (Cention Forte with Cention primer) group had a significantly higher µSBS mean value (26.0360 ± 5.14593 MPa) than all groups (p˂0.05), followed by µSBS mean value of FJI ( Fuji II LC) group (21.7860 ± 3.30991 Mpa) (control group). while the CNF (Cention Forte without Cention primer) group had a significantly lower µSBS mean value (1.5540 ± 1.09871 MPa) than all groups (p˂0.05), the µSBS mean value of SU-O (Surefil One) group (2.1587 ± 1.24608 MPa) was not statistically significant from the value of CNF group (1.5540 ± 1.09871 MPa) (p˃0.05).

For delayed (tested after 6-months of storage in artificial saliva) groups, the CNF + P group had a significantly higher µSBS mean value (21.3773 ± 2.77064 MPa) than all groups (p˂0.05), Contrarily, the difference was not statistically significant (p˃0.05) between the µSBS mean value of CNF group (0.3333 ± 0.86874MPa), SU-O group (0.2060 ± 0.37127MPa) and FJI group (2.2880 ± 1.17500 MPa) (control group).

The results of the current study revealed that aging in artificial saliva had pronounced effect on the micro shear bond strength of some tested restorative material (FJI and CNF + P) as the delayed (tested after 6-months of storage in artificial saliva) group had lower µSBS mean value than the immediate (un-stored) group of the same tested restorative material. The extracted data in this regard revealed that significant difference was found between un-stored and stored groups for some tested materials (p˂0.05).

### Failure mode evaluation

The separation sites of all tested specimens were examined by a stereomicroscope (SZX10; Olympus, Japan) at × 40 magnification and representative samples from each failure mode were gold-coated and examined using a scanning electron microscope (SEM, JSM-6510LV SEM, JEOL Ltd., Tokyo, Japan) at a magnification of approximately 50 × or 55 × to determine the different mode of failure patterns that are showed in Tables 3, 4 explains the predominant failure modes for all groups were adhesive failure. However, the cohesive failure mode percentage was the lowest when compared to other failure modes. The number of adhesive failures was low in immediate group of CNF + P which revealed the highest μSBS mean value. There was no cohesive failure mode recorded among all groups except for CNF + P I and FJI I groups.

**Table 3:** Number of specimens (%) according to fracture Mode and the premature failure of all experimental groups.

Failure mode	Time	Surefil one (%)	Cention forte (%)	Cention forte with primer (%)	Fuji II LC (%)
Adhesive	I	73	87	7	27
	D	33	27	33	87
Cohesive	I	0	0	0	7
	D	0	0	20	0
Mixed	I	27	13	93	67
	D	7	0	47	13
PTF	I	0	0	0	0
	D	60	73	0	0

PTF pretest failure.

**Table 4:** Stereomicroscope and SEM micrographs displaying various failure modes.

Mode of failure	Stereomicroscope images	SEM images
Adhesive		
Cohesive		
Mixed		

### Micromorphological analysis under SEM

Representative SEM micrographs showing the resin-dentin interface at magnifications X1000 of all groups between various restorations. The restoration/dentin interface SEM figures demonstrated that both immediate and delayed group of Cention Forte with Cention primer and Fuji II LC had good, uniform adaption, but in case of Cention Forte without Cention primer and Surefil one groups they showed poor adaption to the underlying dentin.

**Fig. 5:**
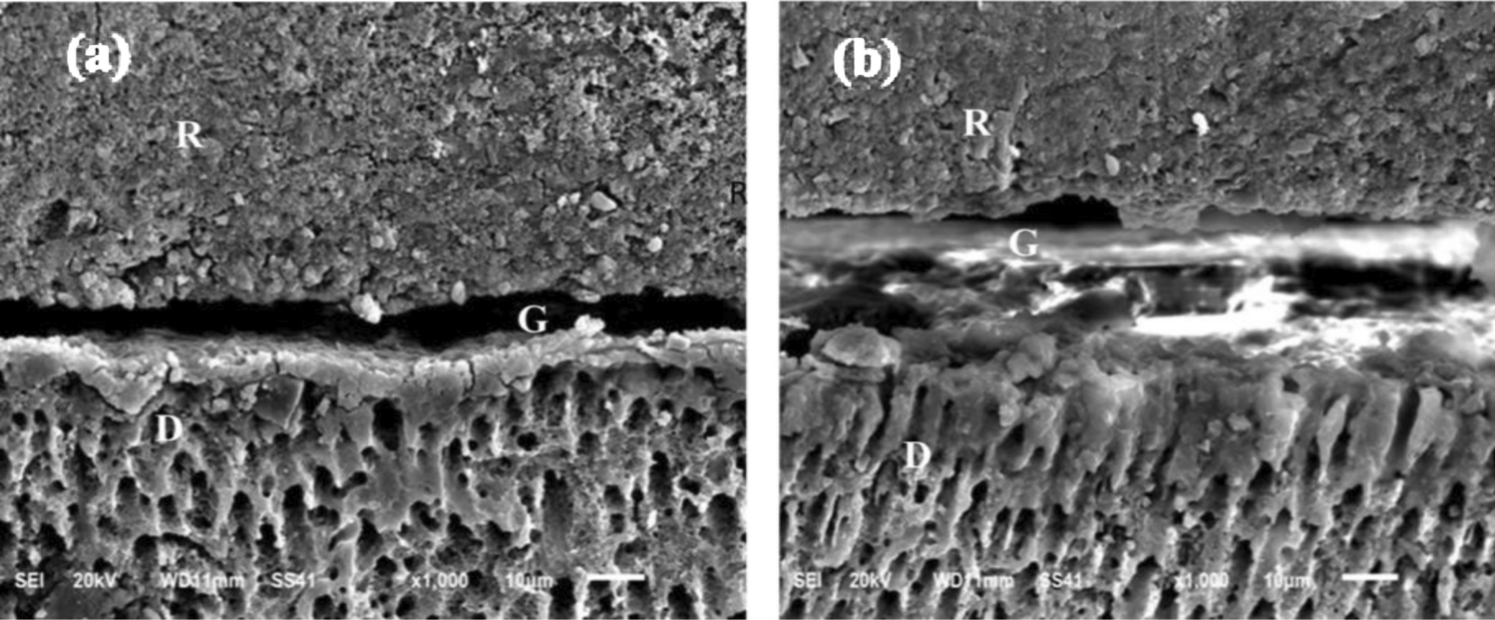
SEM micrographs showing the resin-dentin interface of (1) immediate group and (2) delayed group of surefil one restorative material. Showing in X1000 no resin tags penetrating the dentin surface with evidence of separation and interfacial gaps, there is irregularities at interfacial surface and the smear layer was not completely removed, the dentinal tubules plugging by smear layer, the material/dentin interface appears discontinuous. The separation at the material/dentin interface appear more obvious at delayed specimens. *R* restoration, *D* dentin layer, *G* interfacial gaps.

**Fig. 6:**
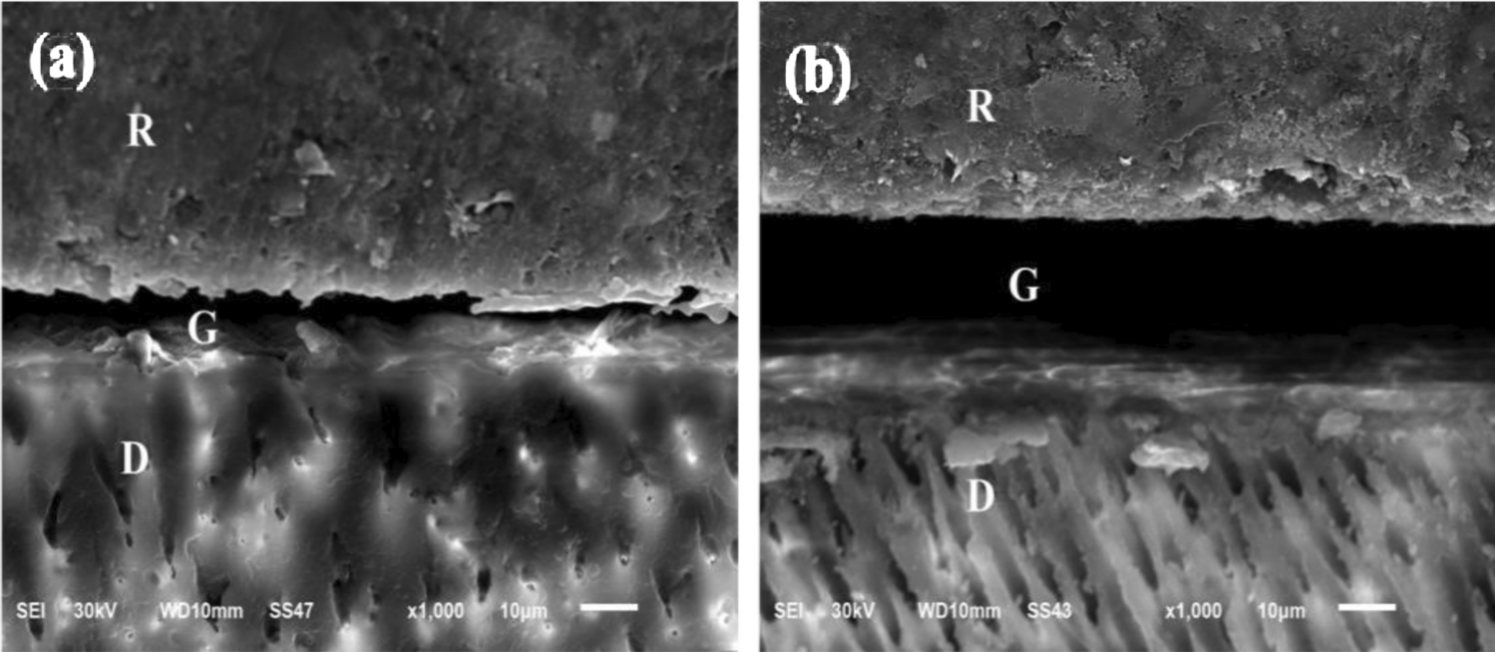
SEM micrographs showing the resin-dentin interface of (1) immediate and (2) delayed group of Cention Forte without Cention primer restorative material. (1) and (2) SEM micrographs in (X1000) magnification.

**Fig. 7:**
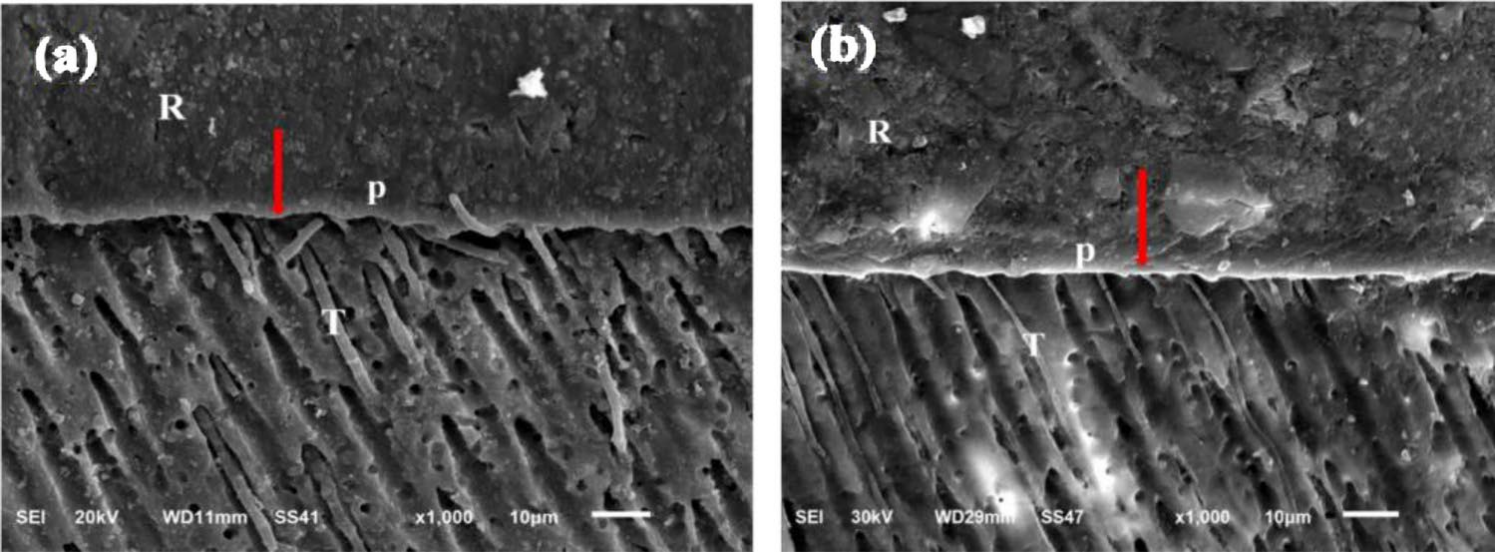
SEM micrographs in (X1000) magnification showing the resin-dentin interface of (1) immediate group and (2) delayed group of Cention Forte with Cention primer restorative material. , (1) show long thick resin tags arranged in a tubular form that penetrate dentin surface, (2) show long and thin resin tags that vary configurations penetrating the dentin interface. Hybrid like layers in reticular patterns was observed. *R* restoration, *P* Cention primer, *D* dentin, *red arrows* hybrid like layer, *T* resin tags.

The SEM micrographs of SU-O & CNF both immediate and delayed groups, the material/dentin interface appears discontinuous. No material is deposited inside dentin tubules. no resin tags penetrating the dentin surface with separation and interfacial gaps, no presence of a hybrid layer , there is irregularities at interfacial surface, the smear layer was not completely removed and the dentinal tubules closed by smear layer plugs as shown in Figs. 5 and 6. The SEM micrographs of CNF + P immediate and delayed groups showed the material/dentin interface appeared to be continuous, long resin tags with broad tubular pattern penetrating the dentin surface with the presence of minor interfacial gaps that represent the previously formed smear layer, and hybrid like layer could be shown in Fig. 7.

**Fig. 8:**
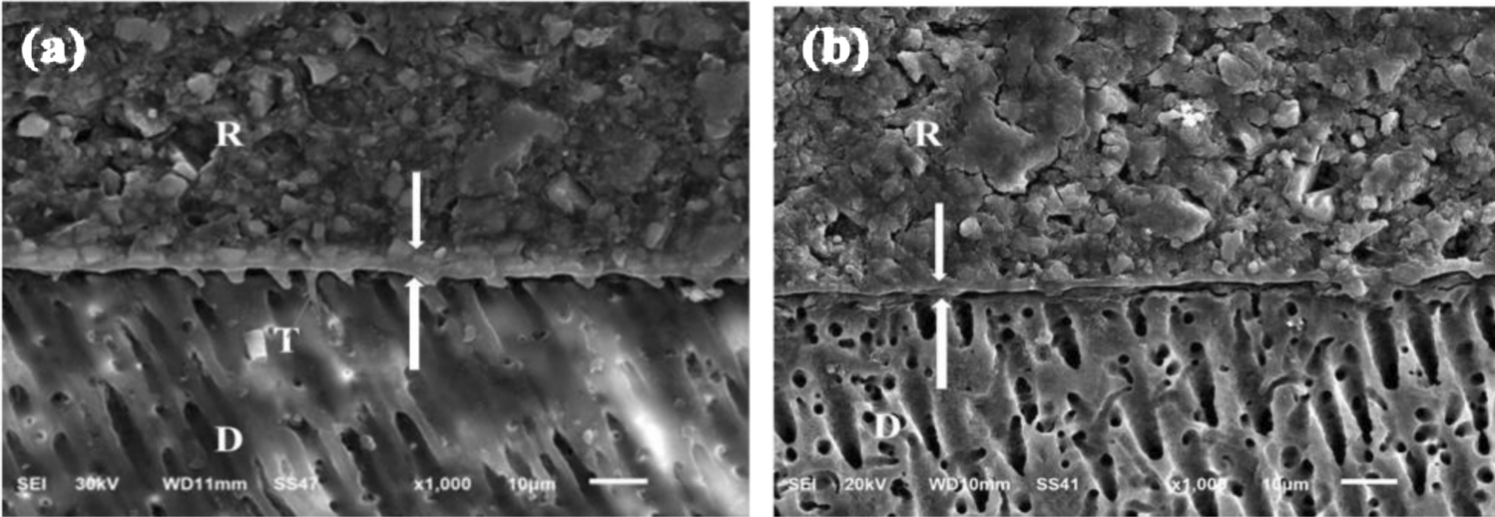
SEM micrographs showing the resin-dentin interface of (1) immediate group and (2) delayed group of Fuji II LC (RMGIC) restorative material. They show budding resin tags penetrating into dentin surface, micrograph (2) show very poor tubular penetration with formation of low budding resin tags magnification. They showing a hybrid like layer and acid base resistance zone formation (white arrows) *R* restoration, *D* dentin, *T* resin tags.

The micromorphological analysis of FJI/pre-conditioned dentin interface revealed that immediate and delayed groups showed complete removal of smear layer with open dentinal tubules created by dentin conditioner, there is intimate contact at the FJI/dentin interface, with budding resin tags and few short and thick resin tags with funnel-shaped pattern penetrating the dentin surface without any evidence of separation or interfacial gaps. The presence of a hybrid like layer was also obvious as shown in Fig. 8.

Showing the material/dentin interface appears discontinuous, hybrid like layer. No material is deposited inside dentin tubules, there is thick smear layer and the dentinal tubules plugging by smear layer, no resin tags penetrating the dentin surface. The separation at the material/dentin interface appear more obvious at delayed specimens. R; restoration, D; dentin G; interfacial gap.

## Discussion

Adhesive interfaces in dental restorations represent a critical area susceptible to weakness. Inadequate adhesion to tooth structure or marginal leakage at this interface can lead to a cascade of undesirable effects, including discoloration, bacterial infiltration, and consequently failure of the restoration^33^. In this respect, the current in-vitro study aimed to assess the bonding performance of two recently introduced self-adhesive bulk-fill hybrid restorative materials compared to a well-established control material RMCIC restoration.

A recently introduced self-adhesive bulk-fill hybrid composite restorative material, was positioned by the manufacturer^34^ as a “forgiving material” that combines the simplified application of a glass-ionomer cement (GIC) with the long-term stability of a conventional resin-based composite (RBC), while maintaining optimal aesthetic outcomes. To objectively assess these claims, the bonding efficacy of self-adhesive bulk-fill hybrid composite on untreated dentin was compared to two established materials: Alkasite-based restorative material and RMGIC. RMGIC served as the control group within the experiment.

The present study evaluated the efficacy of Alkasite-based restorative material, a novel hybrid restorative material, in two application modalities: self-adhesive and non-self-adhesive. Following the manufacturer’s guidelines, Alkasite’s Primer, a dedicated self-adhesive bonding system, was employed to treat the dentin surface in situations involving un retained cavities. However, Alkasite-based restorative material possesses the versatility to function as a self-adhesive material without a primer for retentive cavities.

The resin modified GIC was selected as control material, as it has high affinity to chemically bond to tooth substrate. Furthermore, it is considered as one of the commonly used self-adhesive definitive restorative material. The material was applied following its manufacturer’s instructions after (pre-) conditioning with polyalkenoic-acid (PAA)^35^.

In this study, bonding effectiveness and bond durability after artificial aging (6 months in artificial saliva) were investigated through evaluate 1) microshear bond strength of these tested material, then identify the main failure modes. 2) micromorphological analysis of these materials at the restoration/dentin interface .

The aging procedure is essential in determining the bond durability^36^. In the current study, teeth were stored in artificial saliva in an incubator (BTC, Bio tech company, Cairo, Egypt) at 37 ℃ ± 1 for six months^37^. This was performed to mimic the oral cavity’s environment for assessing the behavior of restorative materials^38^.

A microshear bond strength (μSBS) test was selected to assess bonding strength of the three different restorative materials being applied to flat (mid-coronal) dentin. This test has an essential clinical importance, because the majority of dislodging forces have a shearing effect at the tooth restoration interface^39^. Microshear bons strength testing shows some advantages than the conventional SBS test because small specimens seem to be “stronger” than larger ones. This happens because they are less likely to show critical defects. Nevertheless, if the specimens include some minor defects, they are less likely to be aligned with the force causing the break^40^.

Bond strength to flat dentin in the most ideal condition was compared with bonding in high C-factor class-I cavities in light of a ‘worst-case scenario’, when shrinkage stress was high and severely challenged the bond to cavity-bottom dentin, especially for the restorative materials that are applied in a full self adhesive and bulk-fill application mode (self-adhesive bulk-fill composite, Alkasite-based restorative material)^41^.

Rubber base impression material was identified as the optimal choice for fabricating molds for restorative material micro ‘tygon tubes. This could be attributed to that this fabrication methos does not allow tested material to adhere to testing tube. Therefore this might enhance integrity of microtube structures during mold removing^42,43^.

The outcome of this study revealed that immediate groups showed significant difference in μSBS, except for self-adhesive bulk-fill composite and Alkasite-based restorative material without primer groups that both exhibited a significantly lower bond strength. Upon long-term (6 months) aging in artificial saliva, the aged μSBS of the three restorative material systems decreased significantly as compared to the 24 h immediate μSBS for resin modified GIC, and Alkasite-based restorative material with primer, while no significant decrease in μSBS upon aging was recorded for self-adhesive bulk-fill composite and Alkasite-based restorative material without primer .

The aging had significant effect in this study, because sorption and solubility during storage are common phenomena that lead to chemical changes and adverse effect on the mechanical properties of polymeric material. Based on the composition and microstructure of the materials, the diffusion of solvents into the polymer network causes a volumetric expansion as a result of the separation of polymeric chains. Aqueous solvent absorption causes swelling, which is accompanied by the loss of non-reacted components, erosion of the filler-matrix interface, and plasticization with a decrease in stiffness, hardness, wear resistance, and flexural strength^36^.

Self-adhesive bulk-fill composite exhibited a lower microshear bond strength test results than the pre-treated Alkasite-based restorative material with primer (a competing material) and the immediate group of pre-conditioned RMGIC (control group), there was’t significant difference between self-adhesive bulk-fill composite (immediate and aged) samples. This finding suggests limitations, low in self-adhesive bulk-fill composite’s self-adhesiveness to dentin.

This finding seems contradictory, as self-adhesive bulk-fill composite composition includes features that should promote adhesion:, a high molecular weight polyacrylic acid (intended to promote smear layer hybridization and act as a cross-linking agent for covalent bond formation), Additionally, the material utilizes a hydrolytically stable MOPOS monomer (part of the Modified Polyacid System) that supposedly enhances adhesion to dentin through ionic interactions between dentin’s calcium and MOPOS’ carboxyl groups. Furthermore, MOPOS reportedly functions as a co-polymerizing crosslinker in the cured material. Despite these features, as reported by the manufacturer, self-adhesive bulk-fill composite displayed weak self-adhesion to dentin and did not differ from low microshear bond strength test results Alkasite-based restorative material on untreated dentin^34,44^. Notably, the dominant failure mode in both groups (self-adhesive bulk-fill composite and Alkasite-based restorative material only) was adhesive failure at the dentin interface, it also exhibited a high proportion of pre-test failures (PTF) in delayed groups during storage. Microscopic evaluation of the interface of the alkasite-based restorative material and the self-adhesive hybrid composite with untreated dentin revealed an absence of resin-inter diffusion and signs of separation throughout the interface that represent the smear layer space which correlated with their lower bond strengths. This lack of intimate bonding could be attributed to the presence of a smear layer occluding dentinal tubules and the potential absence of a resin adhesive agent. The notable decrease in μSBS for self-adhesive hybrid composite and alkasite-based-restorative material would imply that these materials might not be able to penetrate the thicker smear layer into the underlying dentin. This furthers support the conclusion that self-adhesive bulk-fill composite’s self-bonding capabilities are limited and the insufficient self-adhesiveness of Alkasite-based restorative material on untreated dentin that was confirmed in this study. In fact, the review by Meerbeek et al. questioned the ability of acidic monomers alone to effect sufficient anchorage to dental tissues^45^.

The insufficient self-adhesiveness of Alkasite-based restorative material on untreated dentin was confirmed in this study, when the significantly lowest immediate and aged μSBS values, no significant different between both and along with a very high incidence of pre-test failures (ptf’s upon aging) was recorded. The present results for Alkasite-based restorative material on un treated dentin are simpler to explain: this material does not contain polyacrylic acid or acidic monomers. These findings support the systematic use of an adhesive system for Alkasite-based restorative material (primer). Previous measurements have shown that specimens without pretreatment had lower SBS than after applying preteatement agent^46^. The pretreatment agents in our study were a dentin conditioner for RMGIC, Primer for Alkasite-based restorative material.

Alkasite-based restorative material is presented with a two-component self-etching and self-curing primer. Alkasite-based restorative material combined with primer generated the highest bond strength to dentin of all tested restorative materials as mean μSBS values were highest for the Alkasite-based restorative material combined with primer in immediate and aged groups (Table 3). No PTF (during specimens’ preparation or during artificial storage) was recorded for the Alkasite-based restorative material combined with primer, suggesting that a separately applied primer appeared to contributed to this better bonding effectiveness, SEM interfacial analysis disclosed the formation of a hybrid layer along with resin tags, indicating primer partially demineralized the dentin surface and inter diffused into partially exposed collagen fibrils.

A high immediate and low aged μSBS values were recorded for the pre-conditioned resin-modified GIC. It’s self-adhesiveness combined micro-mechanical interlocking within submicron hydroxyapatite-rich hybrid-layer formation and primary chemical bonding of carboxylic groups with calcium in hydroxyapatite^47^. The aging process caused a significant decrease in bond strength in RMGIC group, aged RMGIC specimens were recorded very lower μSBS values. There was no PTF (during specimens’ preparation or during artificial storage) recorded for RMGIC group, the mixed failure mode was commonly observed in the immediate sub-group of control group (RMGIC) and the adhesive failure was dominant failure pattern in aged sub-group. The relationship between bond strength and failure modes remains a topic of ongoing investigation.

In the current study the highest value of microshear bond strength (immediate group of alkasite -based restorative material with primer) had low rate of adhesive failure and higher rate of mixed failure, In addition to the lowest microshear bond strength value that related to (delayed group of self-adhesive bulk-fill composite) had a higher rate of adhesive failure. this findings align with Sabatini’s work^48^, which demonstrated a link between the highest bond strength and mixed failure, while the lowest strength corresponded to adhesive failure.

SEM photomicrograph of RMGIC/dentin interface, conditioned with GC conditioner complete removal of smear layer with opened dentinal tubules. There is intimate contact at the RMGIC/dentin interface, with resin few budding resin tags and acid base resistance layer. Acid base resistant layer was observed in all groups of Alkasite-based restorative material combined with primer and RMGIC which is indication of the ability to resist demineralization. Hybrid layer was showed between the dentin and restoration. This support that Alkasite-based restorative material combined with primer possesses ion-releasing property. The bioactivity of the Alkasite-based restorative material and self-adhesive bulk-fill composite has been investigated through several studies^49–51^, showing that the superior rate of Fluoride and calcium ion-releaseand mineralization capabilities of these materials compared to conventional glass ionomer restorations. Previous studies reported a positive correlation between the presence of this hybrid layer and superior bond strengths achieved with RM-GICs compared to other glass ionomer cements (HV-GICs)^52,53^.

The relationship between bond strength and failure modes remains a topic of ongoing investigation. In the current study the highest value of microshear bond strength (immediate group of alkasite -based restorative material with primer) had low rate of adhesive failure and higher rate of mixed failure, In addition to the lowest microshear bond strength value that related to (delayed group of self-adhesive bulk-fill composite) had a higher rate of adhesive failure. this findings align with Sabatini’s work^48^, which demonstrated a link between the highest bond strength and mixed failure, while the lowest strength corresponded to adhesive failure.

In this current study, the micrographs of SEM of each material support the results of μSBS test as the separation at material/dentin interface was shown within materials that has lower mean μSBS values as Cention Forte without Cention primer group and surefil one group. The intemet contact, hybrid like layer and resin tags appeared within the materials that had higher mean μSBS values as Cention Forte with Cention primer group and RMGICs. Overall, in terms of bonding efficacy, the best performing restorative system was alkasite-based material combined with its primer in this study. The groups differed in their interface profiles.

The current findings concur with the research by François et al.^9^ that had reported significant s difference in shear bond strength and interfacial surface formation between self-adhesive bulk-fill composite, alkasite -based restorative material,compared to and high-viscosity glass-ionomer cement (HV-GICs) depending on the material and the dentin pretreatment. SBS values were consistently higher for all materials tested with an adhesive.

Sadeghyar et al.^30^ had investigated the influence of dentin pretreatment on the bond strength of restorative materials. This study agreed with the result of the current study, found that materials requiring dentin pretreatment, such as alkasite -based restorative material with primer, achieve superior bond strength, had almost the double value compared to self-adhesive materials as self-adhesive bulk-fill composite and RMGIC. Furthermore, among materials without pre-treatment, self-adhesive bulk-fill composite exhibits the highest shear bond strength.

Tulesi et al.^54^ evaluated the $\mu$SBS of alkasite-based-restorative material and RM-GIC, Their findings suggested that the superior bond strength achieved with the alkasite material might be attributed to the dentin etching process, the bonding agent to support the use of an adhesive system for alkasite-based-restorative material^55^. This achieved with primer that used in the current study.

On the other hand, resin modified glass-ionomer cements and modern self- adhesive bulk-fill composite restorative materials, showed that they had similar levels of self-adhesiveness to enamel and dentin^24,25^; There is little information available about the bond strength of the self-adhesive bulk-fill restorative.

Based on the results of the current study, the tested null hypothesis that there was no significance difference in microshear bond strength and interfacial micromorphological patterns of three different tested bioactive self-adhesive restorations to dentin, was rejected. In additionally, that the bonding efficacy did not decrease upon substantial artificial aging, was rejected except for the two restorative system mentioned above (alkasite-based material & self-adhesive bulk-fill composite) groups.

Clinical significance of this in-vitro investigation indicate that Alkasite-based material / Primer combination may be a viable substitute for preconditioned Fuji II LC in achieving high bond durability. However, the utilization of Alkasite-based material without its corresponding primer is not recommended. Furthermore, the study does not provide sufficient evidence to support SU-O as an alternative to established permanent restorative materials.

This laboratory study had limitations that extracted teeth lack blood flow, which can affect bonding due to dehydration and collapsed collagen fibers. Additionally, the study couldn’t replicate the dynamic mouth environment with factors like pulpal pressure, fluid flow, and changing pH^56^. Clinical trials are needed to assess longevity of these bioactive restorative materials, how these materials perform in patients. Furthermore, there was a gap in this study on how these hybrid materials release ions and interact with decayed dentin.

## Conclusions

This study revealed that using of primers prior to application of alkasite-based restorative material is highly recommended as this techniques seems to be the most effective in obtaining superior bond strength with dentin. Accordingly, this outcome of this study highlighting the importance of using primer in enhancing bonding to dentin, which might slightly countered the initial manufacturer’s recommendations and categorization of this type of restorations as a self-adhesive.

## Supplementary Information

Supplementary Information 1.

Supplementary Information 2.

## Data Availability

The datasets used and/or analyzed during the current study are available from the corresponding author on reasonable request.
