# Supplementary material for: Evaluation of bond durability of different self-adhesive bioactive restorative systems to dentin
Source: Sci Rep. 2025 Jan 29;15:3667. doi: 10.1038/s41598-024-81351-9 (PMC11779961; doi:10.1038/s41598-024-81351-9)
Supplement: Supplementary file 1 — Supplementary Information 1. [file 41598_2024_81351_MOESM1_ESM.pdf]

Table of raw data of micro-shear bond strength test

Group	Subgroup	**Specimen no**	**μSBS (Mpa)**
Self-adhesive hybrid composite	**Immediate**	1	0.79
		2	2.99
		3	0.22
		4	1.74
		5	3.52
		6	3.36
		7	1.61
		8	2.68
		9	1.3
		10	4.64
		11	3.35
		12	2.07
		13	0.53
		14	2.04
		15	1.54
	**Delayed**	1	0.21
		2	0.13
		3	0.7
		4	1.08
		5	0.92
		6	0.05
		7	0
		8	0
		9	0
		10	0
		11	0
		12	0
		13	0

		14	0
		15	0
**Alkasite-based-restoration** only	Immediate	1	2.65
		2	0.02
		3	1.05
		4	1.61
		5	0.72
		6	3.51
		7	0.82
		8	3.02
		9	2.62
		10	2.86
		11	0.83
		12	1.61
		13	0.66
		14	0.41
		15	0.92
	Delayed	1	3.09
		2	0.05
		3	1.62
		4	0.24
		5	0
		6	0
		7	0
		8	0
		9	0
		10	0
		11	0
		12	0
		13	0
		14	0
		15	0

**Alkasite-based-restoration** with primer	**Immediate**	1	17.02
		2	25.96
		3	17.32
		4	29.29
		5	30.2
		6	22.74
		7	26.07
		8	31.12
		9	33.34
		10	29.3
		11	21.09
		12	24.13
		13	22.48
		14	32.33
		15	28.15
	**Delayed**	1	20.66
		2	22.22
		3	17.74
		4	25.25
		5	15.47
		6	21.8
		7	23.58
		8	24.08
		9	21.33
		10	20.01
		11	22.96
		12	25.03
		13	18.25
		14	19.88
		15	22.4
		1	25.92
		2	19.54

**Resin Modified Glass Ionomer**	**Immediate**	3	23.72
		4	17.83
		5	23.24
		6	17.73
		7	21.96
		8	17.33
		9	20.71
		10	26.79
		11	23.19
		12	22.41
		13	26.88
		14	17.45
		15	22.09
	**Delayed**	1	1.92
		2	3.53
		3	1.84
		4	0.6
		5	3.46
		6	3.87
		7	1.08
		8	1.41
		9	3.11
		10	2.11
		11	2.42
		12	0.95
		13	3.34
		14	3.9
		15	0.78
