# Supplementary material for: Evaluation of bond durability of different self-adhesive bioactive restorative systems to dentin
Source: Sci Rep. 2025 Jan 29;15:3667. doi: 10.1038/s41598-024-81351-9 (PMC11779961; doi:10.1038/s41598-024-81351-9)
Supplement: Supplementary file 2 — Supplementary Information 2. [file 41598_2024_81351_MOESM2_ESM.pdf]

Your temporary usage period for IBM SPSS Statistics will expire in 4319 days.

```
GET
  FILE='C:\Users\2 M TECH\Desktop\raw.sav1.sav'.
DATASET NAME DataSet1 WINDOW=FRONT.
EXAMINE VARIABLES=MSBS BY Groups
  /PLOT BOXPLOT STEMLEAF HISTOGRAM NPPLOT
  /COMPARE GROUPS
  /MESTIMATORS HUBER(1.339) ANDREW(1.34) HAMPEL(1.7,3.4,8.5) TUKEY(4.685)
  /PERCENTILES(5,10,25,50,75,90,95) HAVERAGE
  /STATISTICS DESCRIPTIVES EXTREME
  /CINTERVAL 95
  /MISSING LISTWISE
  /NOTOTAL.
```

Explore

Notes

Output Created		04-MAR-2024 10:06:17
Comments		
Input	Data	C:\Users\2 M TECH\Desktop\raw.sav1.sav
	Active Dataset	DataSet1
	Filter	<none>
	Weight	<none>
	Split File	<none>
	N of Rows in Working Data File	120
Missing Value Handling	Definition of Missing	User-defined missing values for dependent variables are treated as missing.
	Cases Used	Statistics are based on cases with no missing values for any dependent variable or factor used.

Notes

Syntax		EXAMINE VARIABLES=MSBS BY Groups<br>/PLOT BOXPLOT STEMLEAF HISTOGRAM NPPLOT<br>/COMPARE GROUPS<br>/MESTIMATORS HUBER(1.339) ANDREW (1.34) HAMPEL (1.7,3.4,8.5) TUKEY (4.685)<br>/PERCENTILES (5,10,25,50,75,90,95) HAVERAGE<br>/STATISTICS DESCRIPTIVES EXTREME<br>/CINTERVAL 95<br>/MISSING LISTWISE...
Resources	Processor Time	00:00:11.80
	Elapsed Time	00:00:16.45

```
[DataSet1] C:\Users\2 M TECH\Desktop\raw.sav1.sav
```

Groups

Case Processing Summary

		Cases					
		Valid		Missing		Total	
	Groups	N	Percent	N	Percent	N	Percent
MSBS	SI	15	100.0%	0	0.0%	15	100.0%
	SD	15	100.0%	0	0.0%	15	100.0%
	CI	15	100.0%	0	0.0%	15	100.0%
	CD	15	100.0%	0	0.0%	15	100.0%
	CPI	15	100.0%	0	0.0%	15	100.0%
	CPD	15	100.0%	0	0.0%	15	100.0%
	GI	15	100.0%	0	0.0%	15	100.0%
	GD	15	100.0%	0	0.0%	15	100.0%

Descriptives

	Groups			Statistic	Std. Error
MSBS	SI	Mean		2.1587	.32174
		95% Confidence Interval for Mean	Lower Bound	1.4686	
			Upper Bound	2.8487	
		5% Trimmed Mean		2.1285	
		Median		2.0400	
		Variance		1.553	
		Std. Deviation		1.24608	
		Minimum		.22	
		Maximum		4.64	
		Range		4.42	
		Interquartile Range		2.05	
		Skewness		.284	.580
		Kurtosis		-.511	1.121
	SD	Mean		.2060	.09586
		95% Confidence Interval for Mean	Lower Bound	.0004	
			Upper Bound	.4116	
		5% Trimmed Mean		.1689	
		Median		.0000	
		Variance		.138	
		Std. Deviation		.37127	
		Minimum		.00	
		Maximum		1.08	
		Range		1.08	
		Interquartile Range		.21	
		Skewness		1.722	.580
		Kurtosis		1.551	1.121
	CI	Mean		1.5540	.28368
		95% Confidence Interval for Mean	Lower Bound	.9456	
			Upper Bound	2.1624	
		5% Trimmed Mean		1.5306	
		Median		1.0500	
		Variance		1.207	
		Std. Deviation		1.09871	
		Minimum		.02	
		Maximum		3.51	

Descriptives

Groups				Statistic	Std. Error
		Range		3.49	
		Interquartile Range		1.93	
		Skewness		.487	.580
		Kurtosis		-1.200	1.121
	CD	Mean		.3333	.22431
		95% Confidence Interval for Mean	Lower Bound	-.1478	
			Upper Bound	.8144	
		5% Trimmed Mean		.1987	
		Median		.0000	
		Variance		.755	
		Std. Deviation		.86874	
		Minimum		.00	
		Maximum		3.09	
		Range		3.09	
		Interquartile Range		.05	
		Skewness		2.847	.580
		Kurtosis		7.928	1.121
	CPI	Mean		26.0360	1.32867
		95% Confidence Interval for Mean	Lower Bound	23.1863	
			Upper Bound	28.8857	
		5% Trimmed Mean		26.1311	
		Median		26.0700	
		Variance		26.481	
		Std. Deviation		5.14593	
		Minimum		17.02	
		Maximum		33.34	
		Range		16.32	
		Interquartile Range		7.72	
		Skewness		-.413	.580
		Kurtosis		-.808	1.121
	CPD	Mean		21.3773	.71538
		95% Confidence Interval for Mean	Lower Bound	19.8430	
			Upper Bound	22.9117	
		5% Trimmed Mean		21.4904	
		Median		21.8000	

Descriptives

Groups				Statistic	Std. Error
		Variance		7.676	
		Std. Deviation		2.77064	
		Minimum		15.47	
		Maximum		25.25	
		Range		9.78	
		Interquartile Range		3.70	
		Skewness		-.574	.580
		Kurtosis		-.068	1.121
	GI	Mean		21.7860	.85461
		95% Confidence Interval for Mean	Lower Bound	19.9530	
			Upper Bound	23.6190	
		5% Trimmed Mean		21.7506	
		Median		22.0900	
		Variance		10.955	
		Std. Deviation		3.30991	
		Minimum		17.33	
		Maximum		26.88	
		Range		9.55	
		Interquartile Range		5.89	
		Skewness		.069	.580
		Kurtosis		-1.109	1.121
	GD	Mean		2.2880	.30338
		95% Confidence Interval for Mean	Lower Bound	1.6373	
			Upper Bound	2.9387	
		5% Trimmed Mean		2.2922	
		Median		2.1100	
		Variance		1.381	
		Std. Deviation		1.17500	
		Minimum		.60	
		Maximum		3.90	
		Range		3.30	
		Interquartile Range		2.38	
		Skewness		.009	.580
		Kurtosis		-1.565	1.121

M-Estimators[e]

	Groups	Huber's M-Estimator[a]	Tukey's Biweight[b]	Hampel's M-Estimator[c]	Andrews' Wave[d]
MSBS	SI	2.1383	2.1016	2.1161	2.1021
	SD	.	.	.	.
	CI	1.3062	1.1734	1.3730	1.1603
	CD	.	.	.	.
	CPI	26.4329	26.4489	26.3869	26.4446
	CPD	21.6605	21.7649	21.6084	21.7657
	GI	21.7471	21.7807	21.6949	21.7815
	GD	2.2908	2.2858	2.2880	2.2858

a. The weighting constant is 1.339.

b. The weighting constant is 4.685.

c. The weighting constants are 1.700, 3.400, and 8.500

d. The weighting constant is 1.340*pi.

e. Some M-Estimators cannot be computed because of the highly centralized distribution around the median.

Percentiles

			Percentiles				
		Groups	5	10	25	50	75
Weighted Average (Definition 1)	MSBS	SI	.2200	.4060	1.3000	2.0400	3.3500
		SD	.0000	.0000	.0000	.0000	.2100
		CI	.0200	.2540	.7200	1.0500	2.6500
		CD	.0000	.0000	.0000	.0000	.0500
		CPI	17.0200	17.2000	22.4800	26.0700	30.2000
		CPD	15.4700	16.8320	19.8800	21.8000	23.5800
		GI	17.3300	17.4020	17.8300	22.0900	23.7200
		GD	.6000	.7080	1.0800	2.1100	3.4600
Tukey's Hinges	MSBS	SI			1.4200	2.0400	3.1700
		SD			.0000	.0000	.1700
		CI			.7700	1.0500	2.6350
		CD			.0000	.0000	.0250
		CPI			22.6100	26.0700	29.7500
		CPD			19.9450	21.8000	23.2700
		GI			18.6850	22.0900	23.4800
		GD			1.2450	2.1100	3.4000

Percentiles

		Groups	Percentiles 90	95
Weighted Average (Definition 1)	MSBS	SI	3.9680	.
		SD	.9840	.
		CI	3.2160	.
		CD	2.2080	.
		CPI	32.7340	.
		CPD	25.1180	.
		GI	26.8260	.
		GD	3.8820	.
Tukey's Hinges	MSBS	SI		
		SD		
		CI		
		CD		
		CPI		
		CPD		
		GI		
		GD		

Extreme Values

Groups				Case Number	Value
MSBS	SI	Highest	1	10	4.64
			2	5	3.52
			3	6	3.36
			4	11	3.35
			5	2	2.99
		Lowest	1	3	.22
			2	13	.53
			3	1	.79
			4	9	1.30
			5	15	1.54
	SD	Highest	1	19	1.08
			2	20	.92
			3	18	.70
			4	16	.21
			5	17	.13
		Lowest	1	30	.00
			2	29	.00
			3	28	.00
			4	27	.00
			5	26	.00[a]
	CI	Highest	1	36	3.51
			2	38	3.02
			3	40	2.86
			4	31	2.65
			5	39	2.62
		Lowest	1	32	.02
			2	44	.41
			3	43	.66
			4	35	.72
			5	37	.82
	CD	Highest	1	46	3.09
			2	48	1.62

Extreme Values

Groups			Case Number	Value
		3	49	.24
		4	47	.05
		5	50	.00[b]
	Lowest	1	60	.00
		2	59	.00
		3	58	.00
		4	57	.00
		5	56	.00[a]
CPI	Highest	1	69	33.34
		2	74	32.33
		3	68	31.12
		4	65	30.20
		5	70	29.30
	Lowest	1	61	17.02
		2	63	17.32
		3	71	21.09
		4	73	22.48
		5	66	22.74
CPD	Highest	1	79	25.25
		2	87	25.03
		3	83	24.08
		4	82	23.58
		5	86	22.96
	Lowest	1	80	15.47
		2	78	17.74
		3	88	18.25
		4	89	19.88
		5	85	20.01
GI	Highest	1	103	26.88
		2	100	26.79
		3	91	25.92
		4	93	23.72
		5	95	23.24

Extreme Values

Groups			Case Number	Value
	Lowest	1	98	17.33
		2	104	17.45
		3	96	17.73
		4	94	17.83
		5	92	19.54
GD	Highest	1	119	3.90
		2	111	3.87
		3	107	3.53
		4	110	3.46
		5	118	3.34
	Lowest	1	109	.60
		2	120	.78
		3	117	.95
		4	112	1.08
		5	113	1.41

a. Only a partial list of cases with the value .00 are shown in the table of lower extremes.

b. Only a partial list of cases with the value .00 are shown in the table of upper extremes.

Tests of Normality

		Kolmogorov-Smirnov[a]			Shapiro-Wilk		
	Groups	Statistic	df	Sig.	Statistic	df	Sig.
MSBS	SI	.128	15	.200*	.972	15	.881
	SD	.329	15	.000	.623	15	.000
	CI	.210	15	.074	.906	15	.119
	CD	.428	15	.000	.457	15	.000
	CPI	.136	15	.200*	.949	15	.510
	CPD	.094	15	.200*	.966	15	.789
	GI	.151	15	.200*	.922	15	.209
	GD	.158	15	.200*	.916	15	.164

*. This is a lower bound of the true significance.

a. Lilliefors Significance Correction

MSBS

Histograms

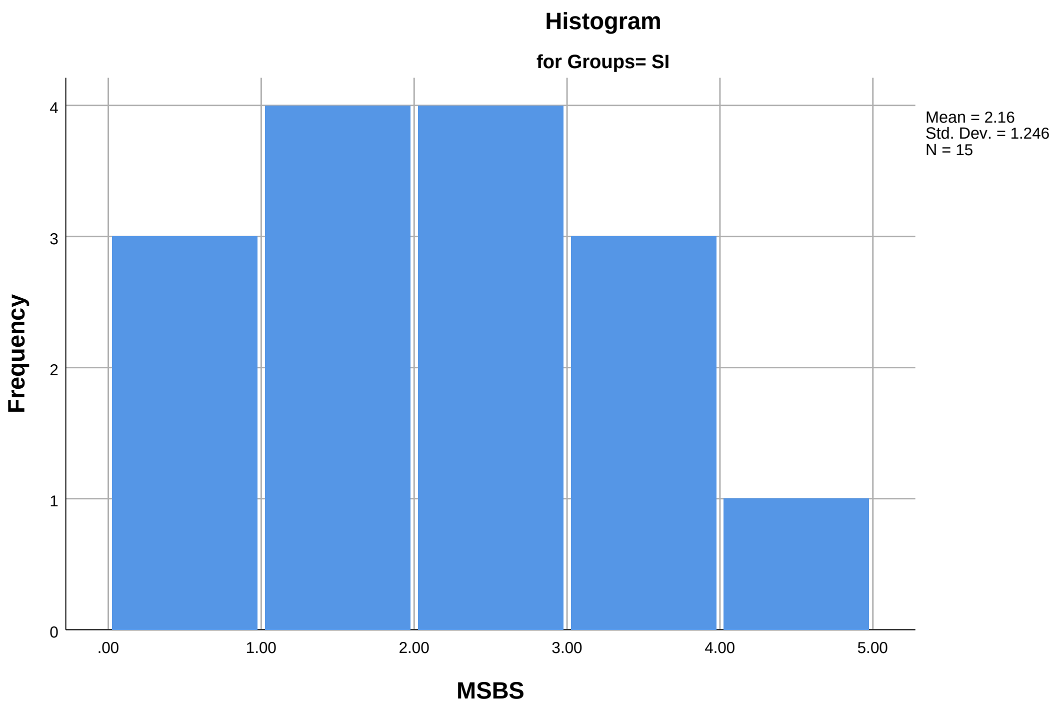

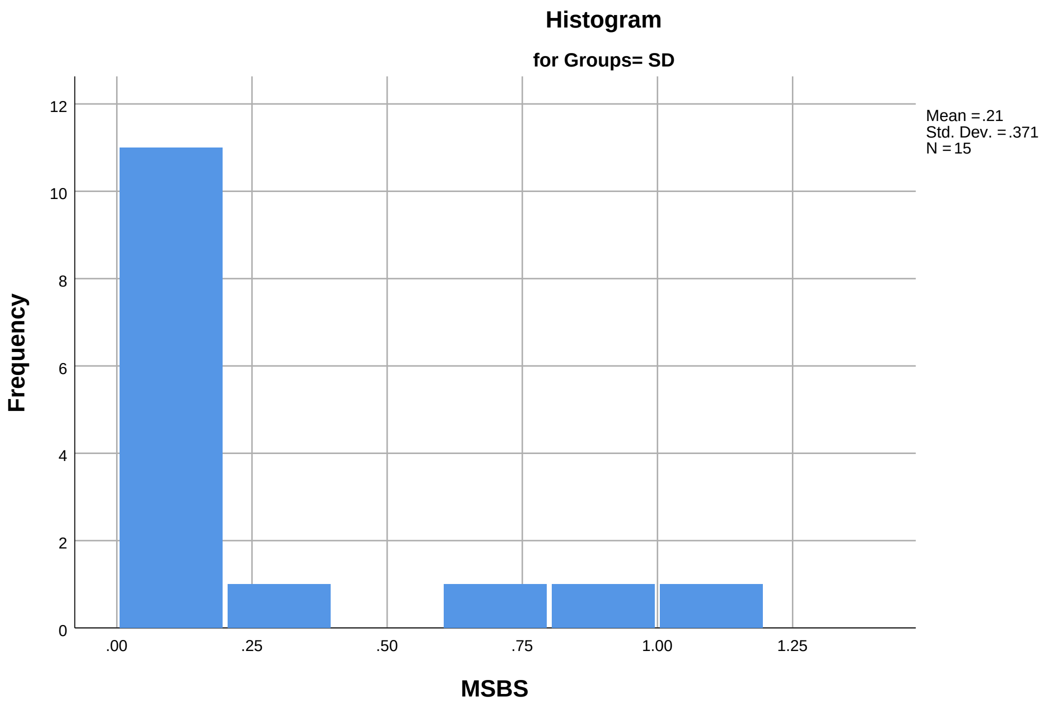

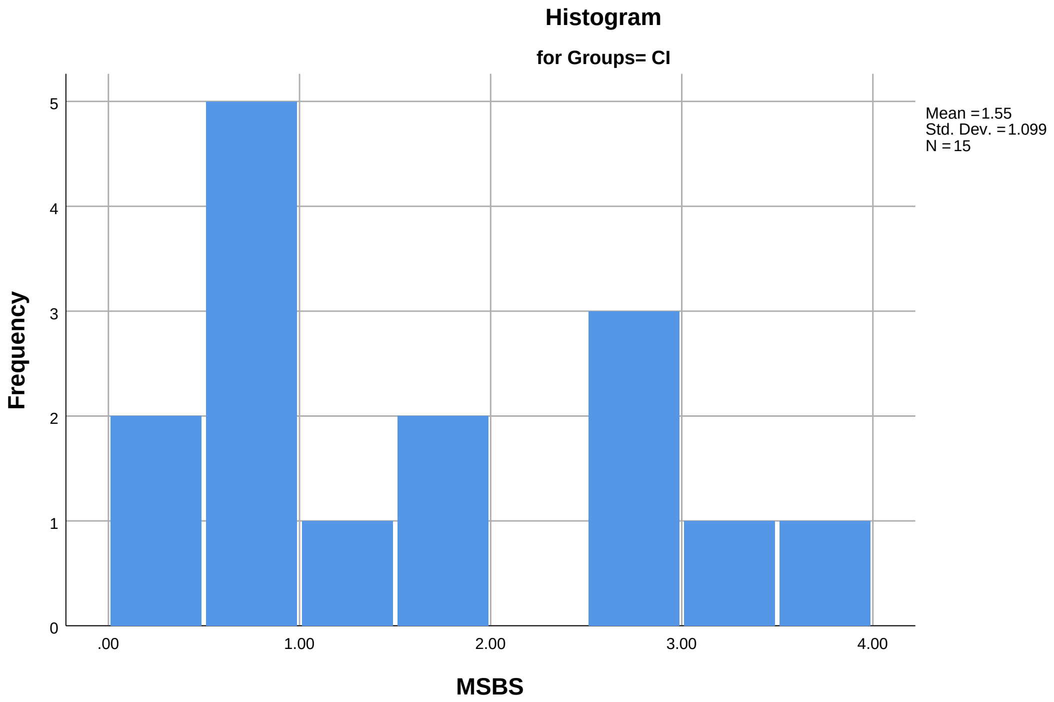
Histogram
for Groups= CI
Mean =1.55
Std. Dev. =1.099
N =15
Frequency
0
1
2
3
4
5
.00
1.00
2.00
3.00
4.00
MSBS

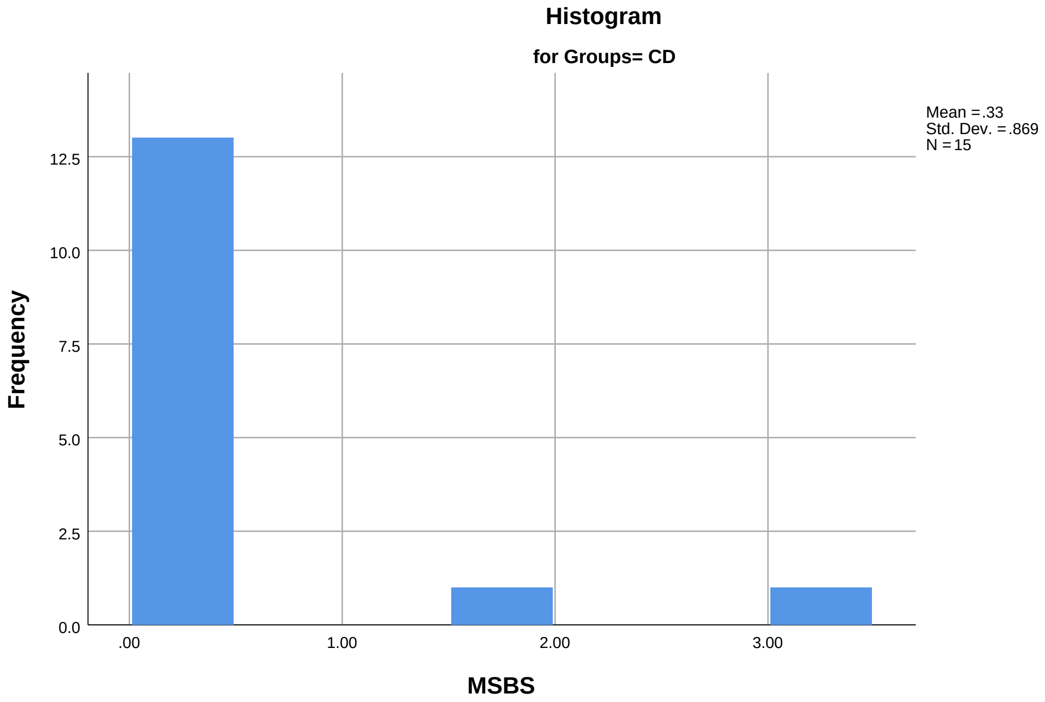
Histogram
for Groups= CD
Mean =.33
Std. Dev. =.869
N =15
Frequency
0.0
2.5
5.0
7.5
10.0
12.5
.00
1.00
2.00
3.00
MSBS

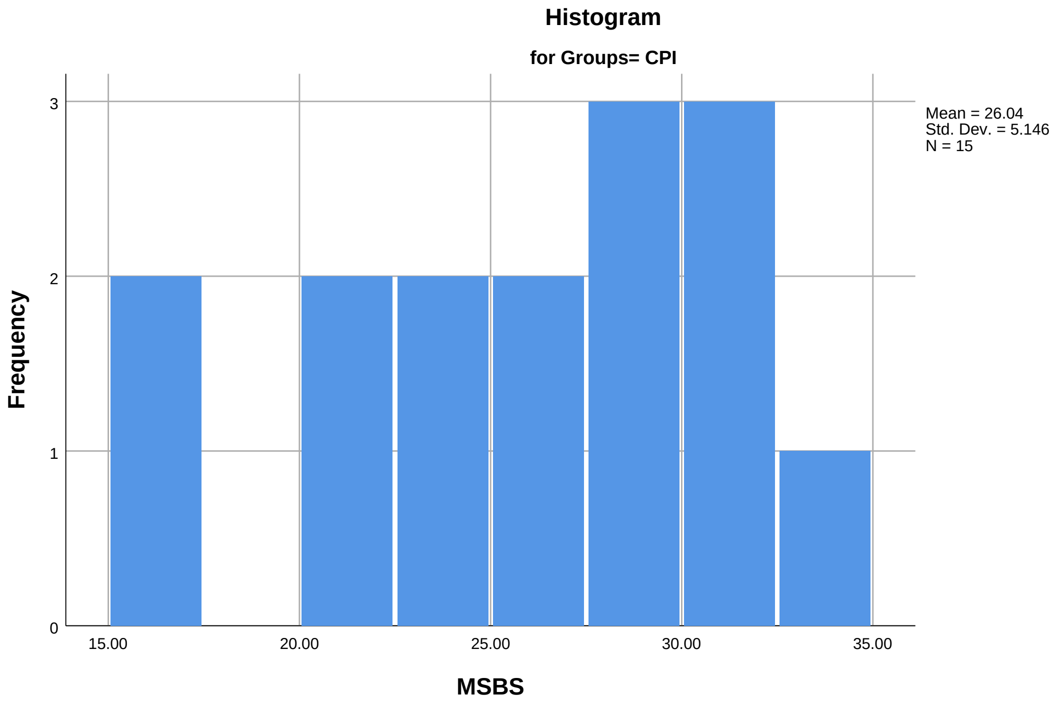
Histogram
for Groups= CPI
Mean = 26.04
Std. Dev. = 5.146
N = 15
Frequency
0
1
2
3
15.00
20.00
25.00
30.00
35.00
MSBS

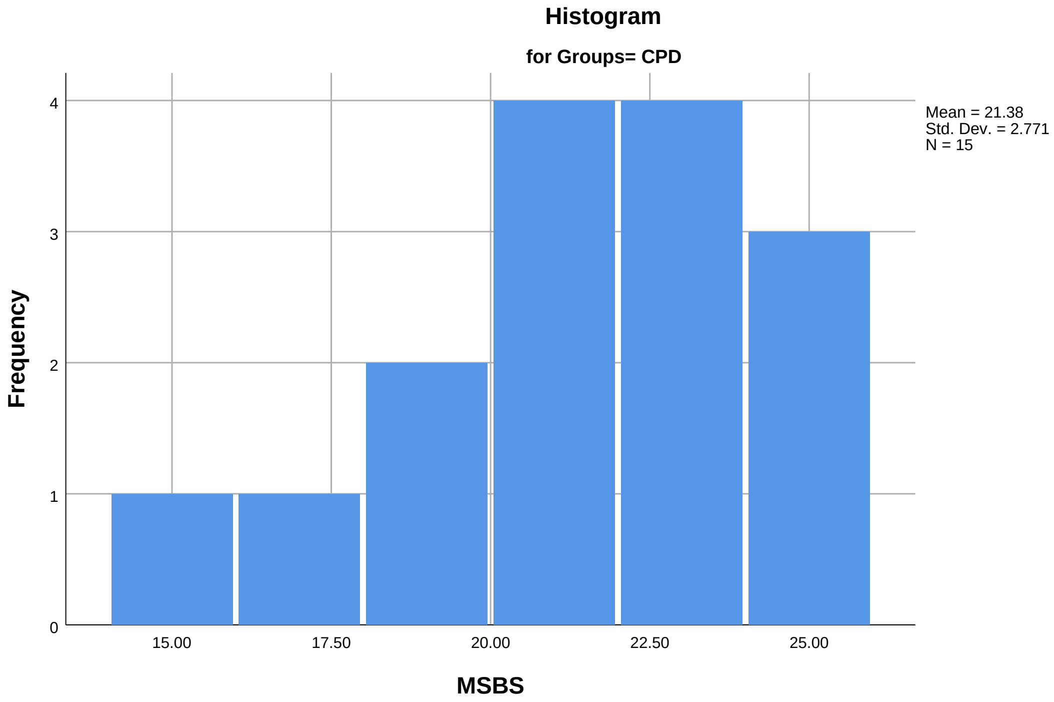
Histogram
for Groups= CPD
Mean = 21.38
Std. Dev. = 2.771
N = 15
Frequency
0
1
2
3
4
15.00
17.50
20.00
22.50
25.00
MSBS

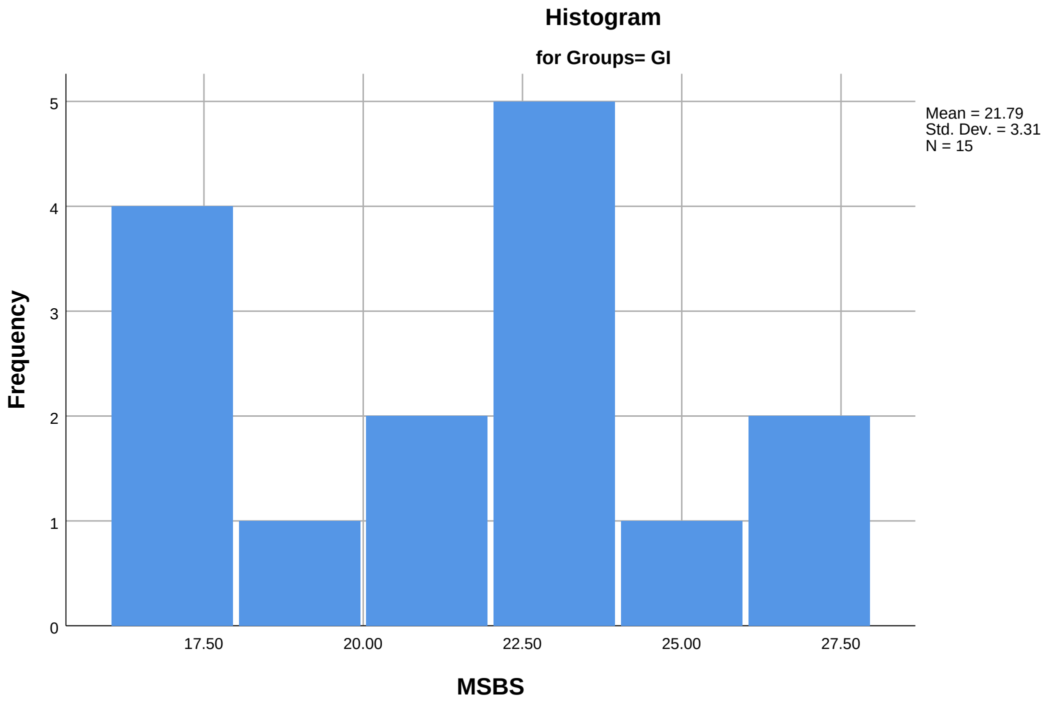

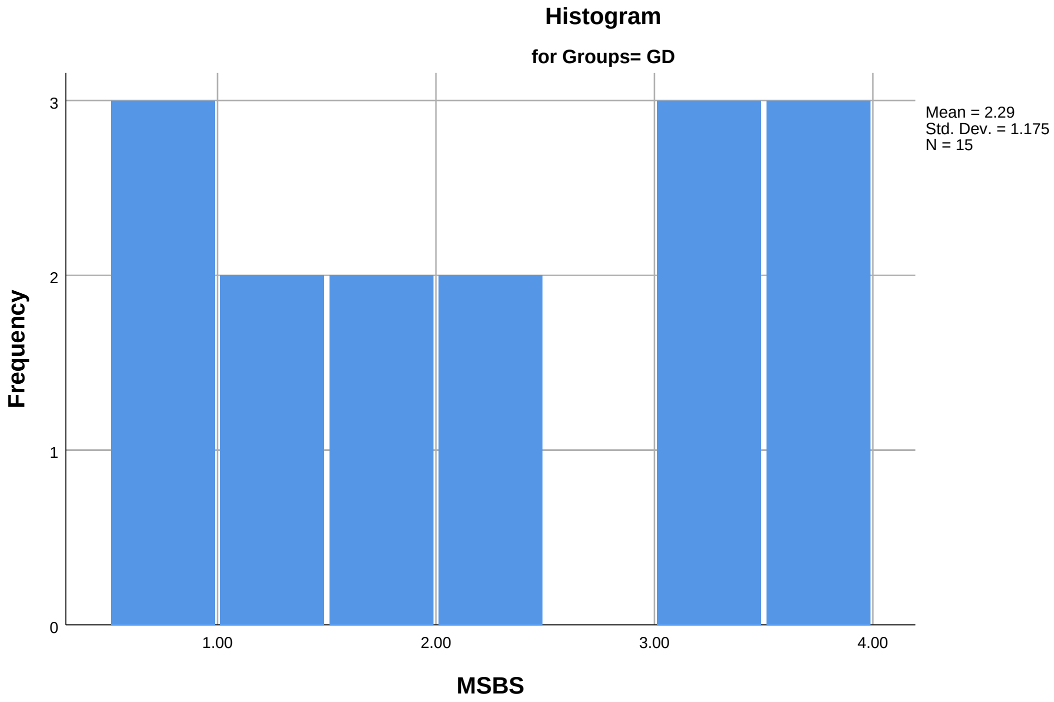

Stem-and-Leaf Plots

```
MSBS Stem-and-Leaf Plot for
Groups= SI
```

```
 Frequency    Stem &  Leaf

     3.00        0 .  257
     4.00        1 .  3567
     4.00        2 .  0069
     3.00        3 .  335
     1.00        4 .  6

 Stem width:      1.00
 Each leaf:        1 case(s)
```

```
MSBS Stem-and-Leaf Plot for
Groups= SD

 Frequency    Stem &  Leaf

     9.00        0 .  000000000
     1.00        0 .  5
     1.00        1 .  3
      .00        1 .
     1.00        2 .  1
     3.00 Extremes    (>=.70)

 Stem width:       .10
 Each leaf:        1 case(s)
```

```
MSBS Stem-and-Leaf Plot for
Groups= CI

 Frequency    Stem &  Leaf

     7.00        0 .  0467889
     3.00        1 .  066
     3.00        2 .  668
     2.00        3 .  05

 Stem width:      1.00
```

```
 Each leaf:        1 case(s)



MSBS Stem-and-Leaf Plot for
Groups= CD

 Frequency    Stem &  Leaf

    11.00        0 .  00000000000
      .00        1 .
      .00        2 .
      .00        3 .
      .00        4 .
     1.00        5 .  0
     3.00 Extremes    (>=.240)

 Stem width:       .01
 Each leaf:        1 case(s)



MSBS Stem-and-Leaf Plot for
Groups= CPI

 Frequency    Stem &  Leaf

     2.00        1 .  77
     4.00        2 .  1224
     5.00        2 .  56899
     4.00        3 .  0123

 Stem width:     10.00
 Each leaf:        1 case(s)



MSBS Stem-and-Leaf Plot for
Groups= CPD

 Frequency    Stem &  Leaf

     4.00        1 .  5789
```

```
     9.00        2 .  001122234
     2.00        2 .  55

 Stem width:     10.00
 Each leaf:        1 case(s)
```

```
MSBS Stem-and-Leaf Plot for
Groups= GI

 Frequency    Stem &  Leaf

     5.00        1 .  77779
     7.00        2 .  0122333
     3.00        2 .  566

 Stem width:     10.00
 Each leaf:        1 case(s)
```

```
MSBS Stem-and-Leaf Plot for
Groups= GD

 Frequency    Stem &  Leaf

     3.00        0 .  679
     4.00        1 .  0489
     2.00        2 .  14
     6.00        3 .  134589

 Stem width:      1.00
 Each leaf:        1 case(s)
```

Normal Q-Q Plots

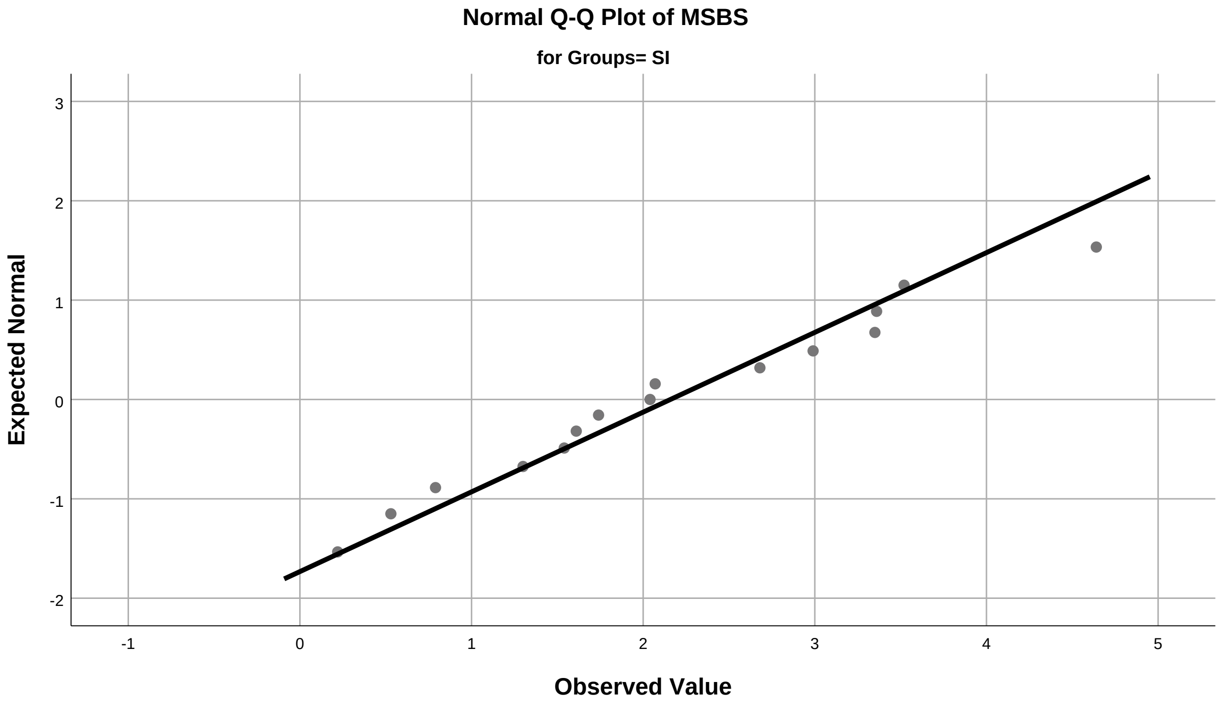
Normal Q-Q Plot of MSBS
for Groups= SI
Expected Normal
Observed Value
3
2
1
0
-1
-2
-1
0
1
2
3
4
5

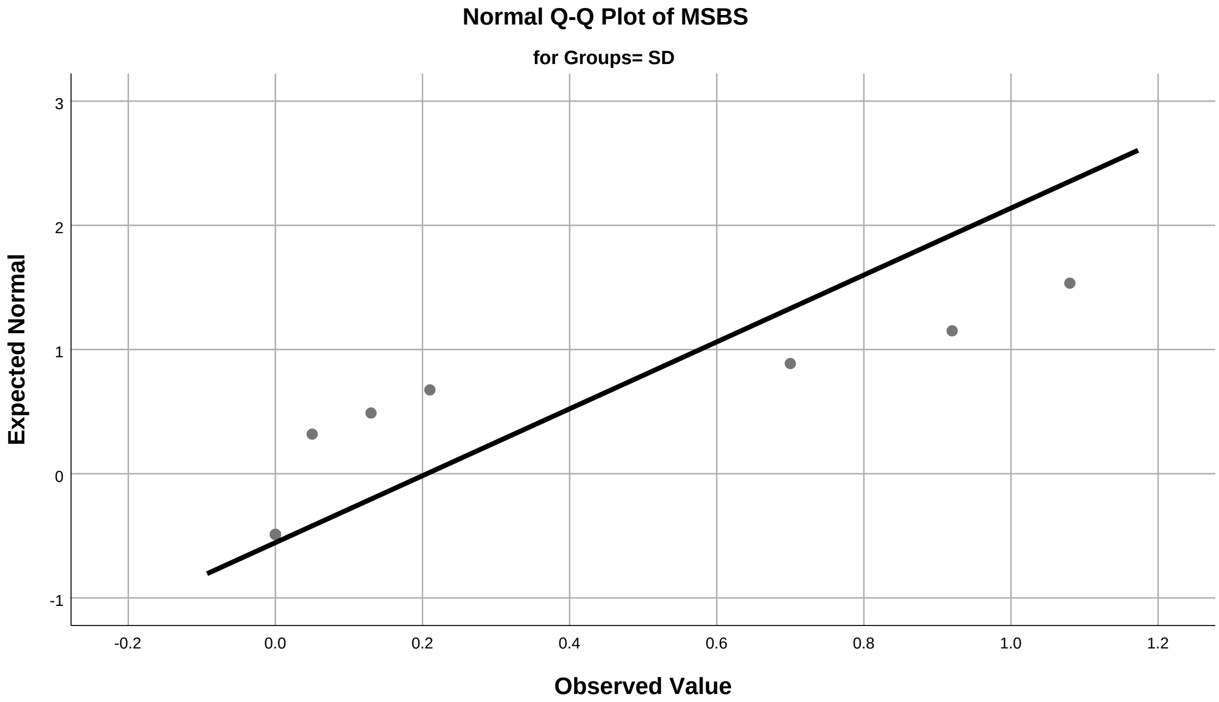
Normal Q-Q Plot of MSBS
for Groups= SD
Expected Normal
Observed Value
3
2
1
0
-1
-0.2
0.0
0.2
0.4
0.6
0.8
1.0
1.2

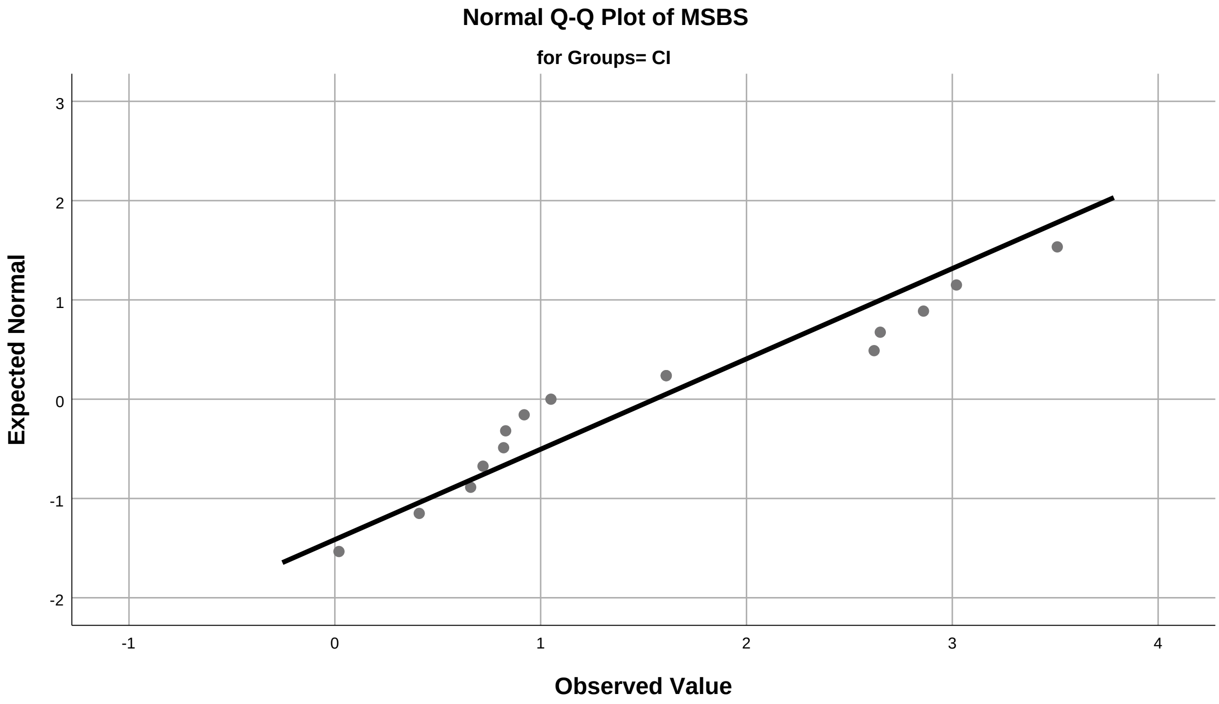
Normal Q-Q Plot of MSBS
for Groups= CI
Expected Normal
3
2
1
0
-1
-2
-1
0
1
2
3
4
Observed Value

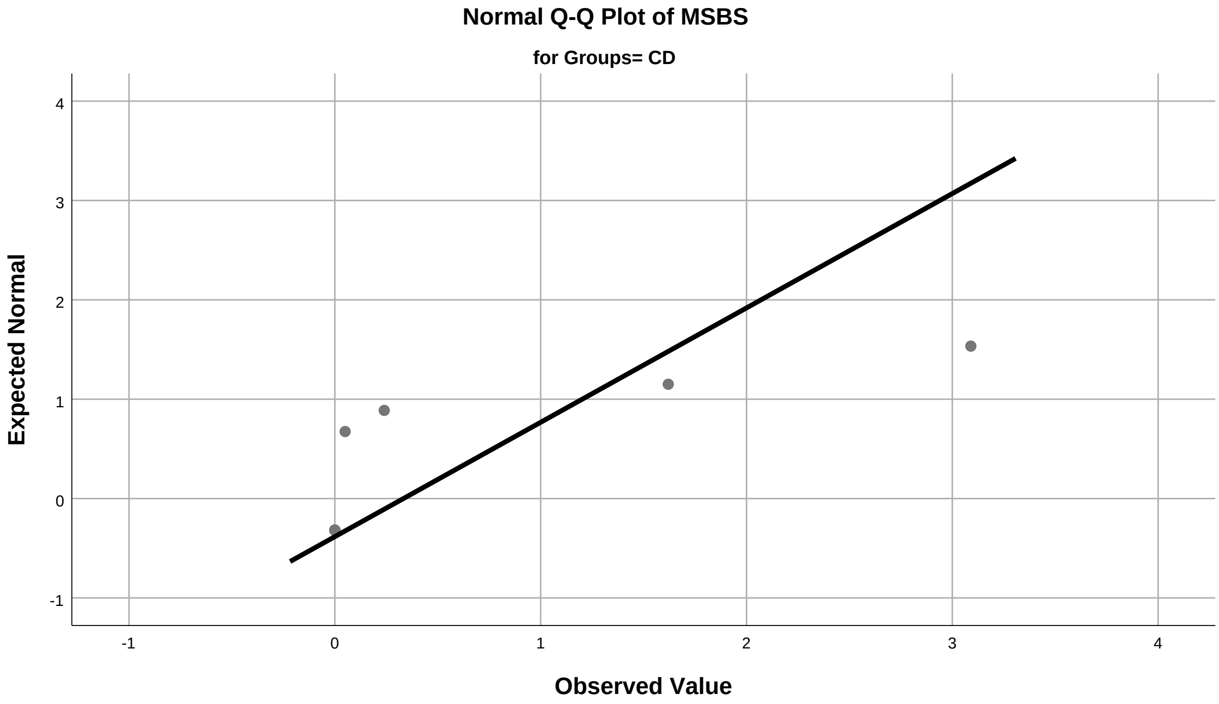
Normal Q-Q Plot of MSBS
for Groups= CD
Expected Normal
4
3
2
1
0
-1
-1
0
1
2
3
4
Observed Value

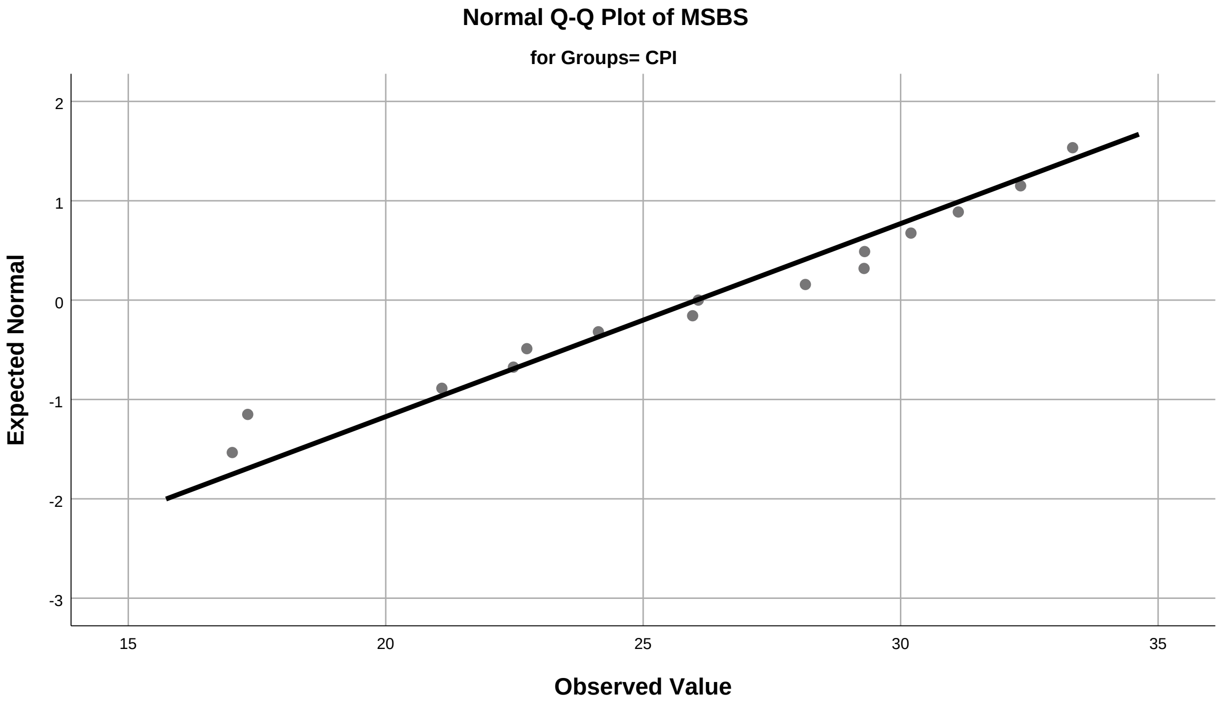
Normal Q-Q Plot of MSBS
for Groups= CPI
Expected Normal
Observed Value
2
1
0
-1
-2
-3
15
20
25
30
35

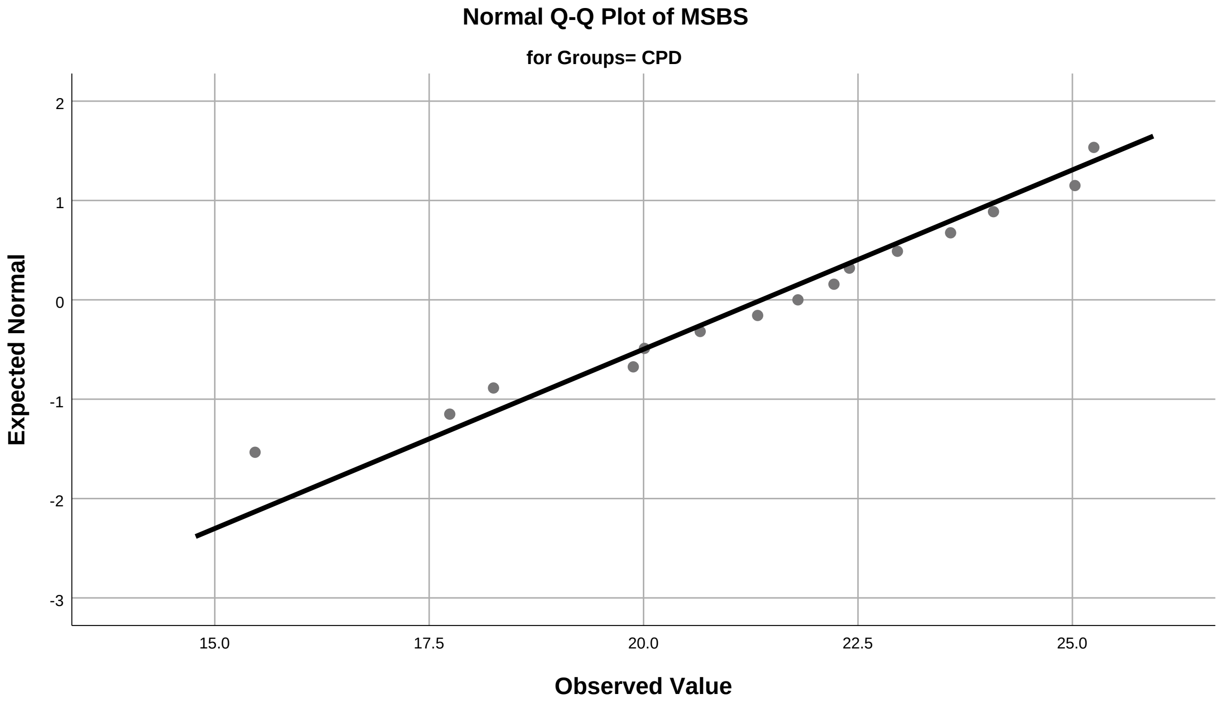
Normal Q-Q Plot of MSBS
for Groups= CPD
Expected Normal
Observed Value
2
1
0
-1
-2
-3
15.0
17.5
20.0
22.5
25.0

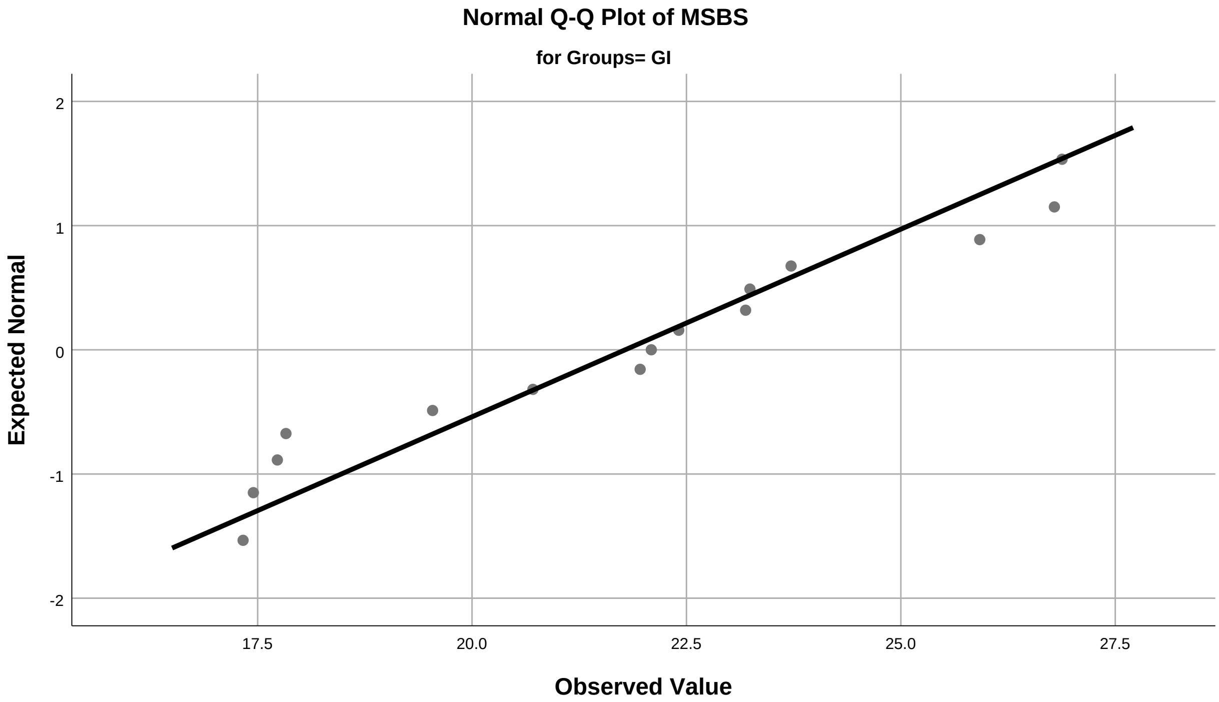

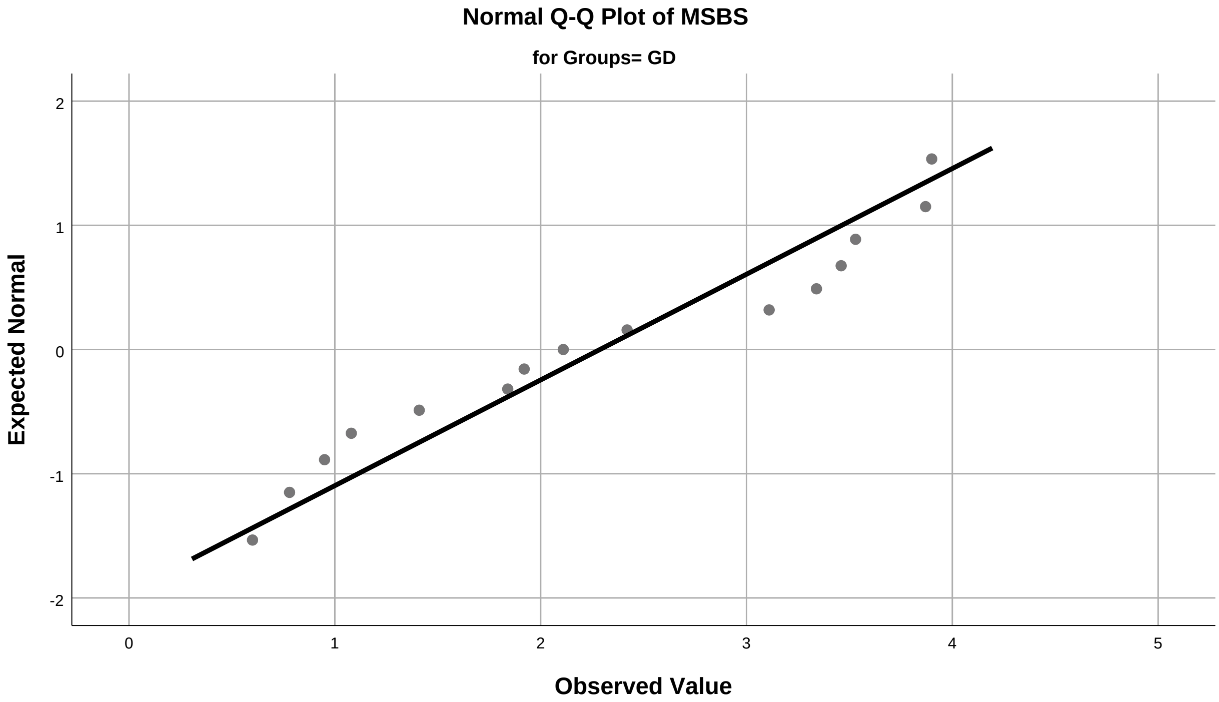

Detrended Normal Q-Q Plots

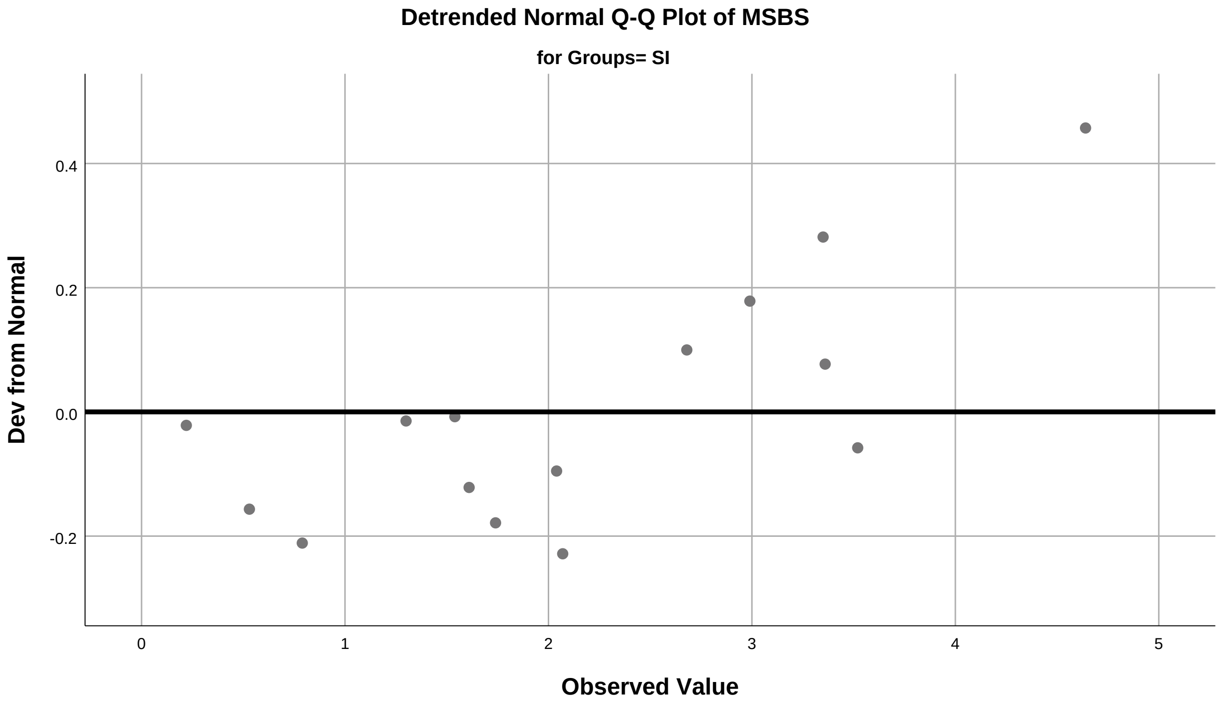
Detrended Normal Q-Q Plot of MSBS
for Groups= SI
Dev from Normal
0.4
0.2
0.0
-0.2
0
1
2
3
4
5
Observed Value

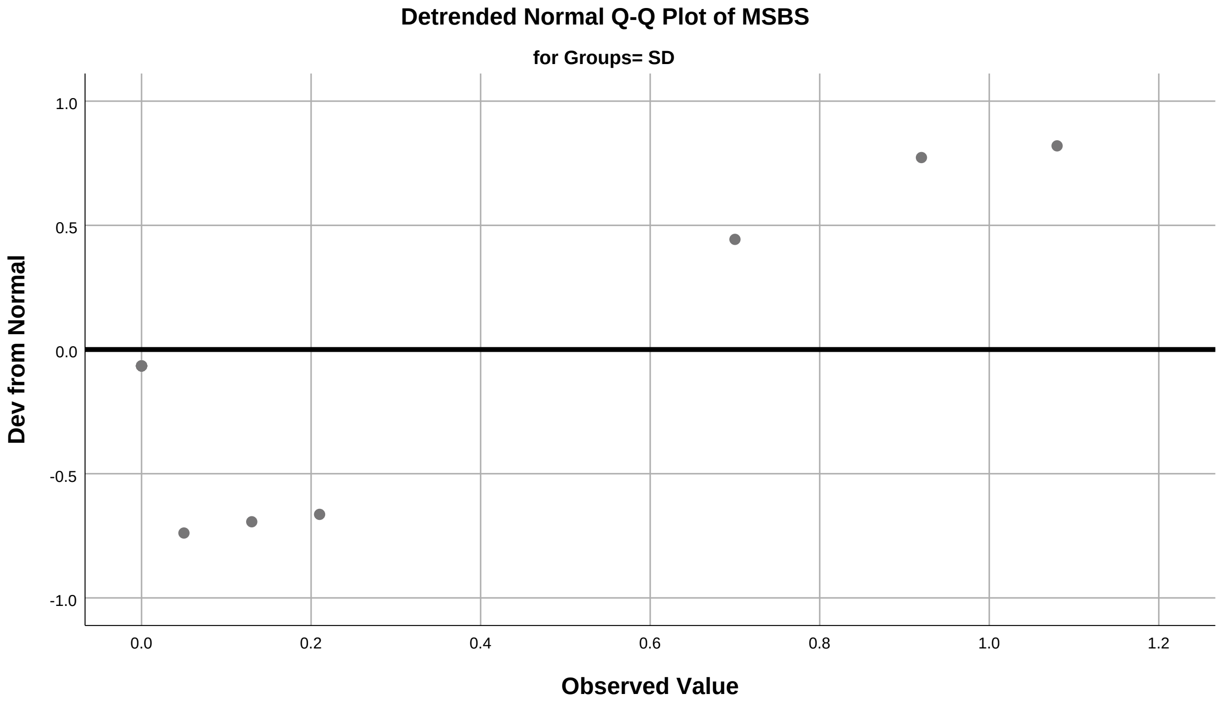
Detrended Normal Q-Q Plot of MSBS
for Groups= SD
Dev from Normal
1.0
0.5
0.0
-0.5
-1.0
0.0
0.2
0.4
0.6
0.8
1.0
1.2
Observed Value

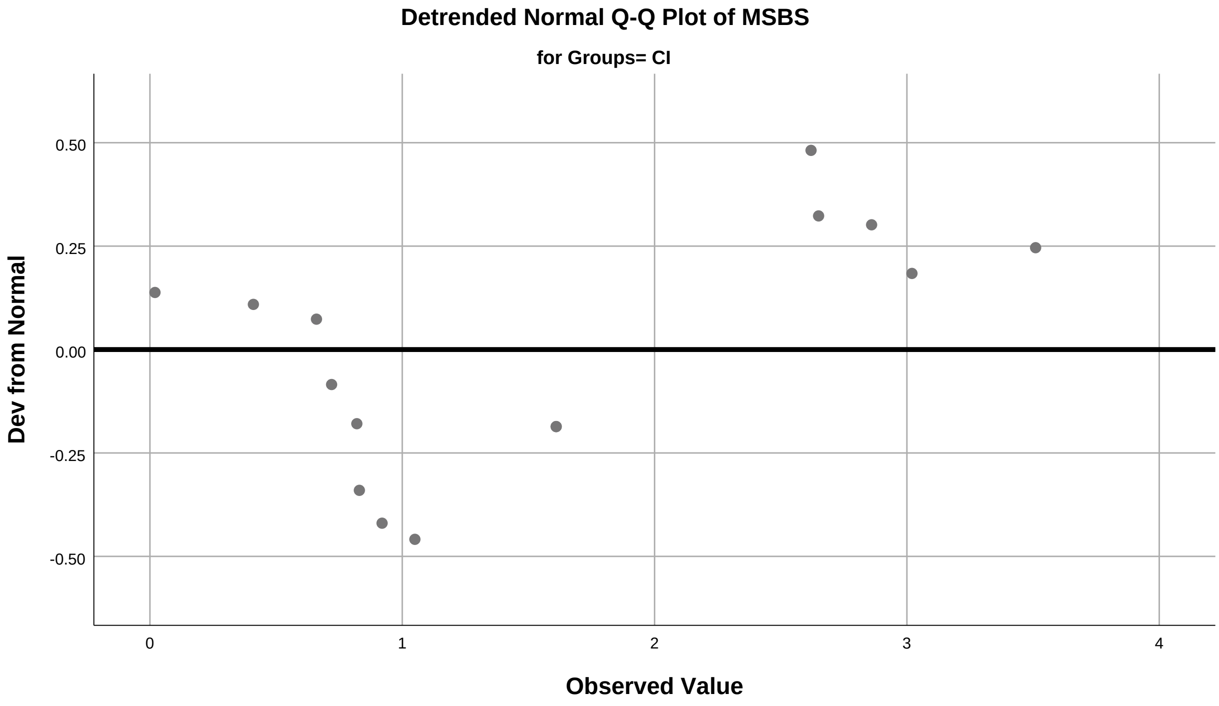
Detrended Normal Q-Q Plot of MSBS
for Groups= CI
Dev from Normal
0.50
0.25
0.00
-0.25
-0.50
0
1
2
3
4
Observed Value

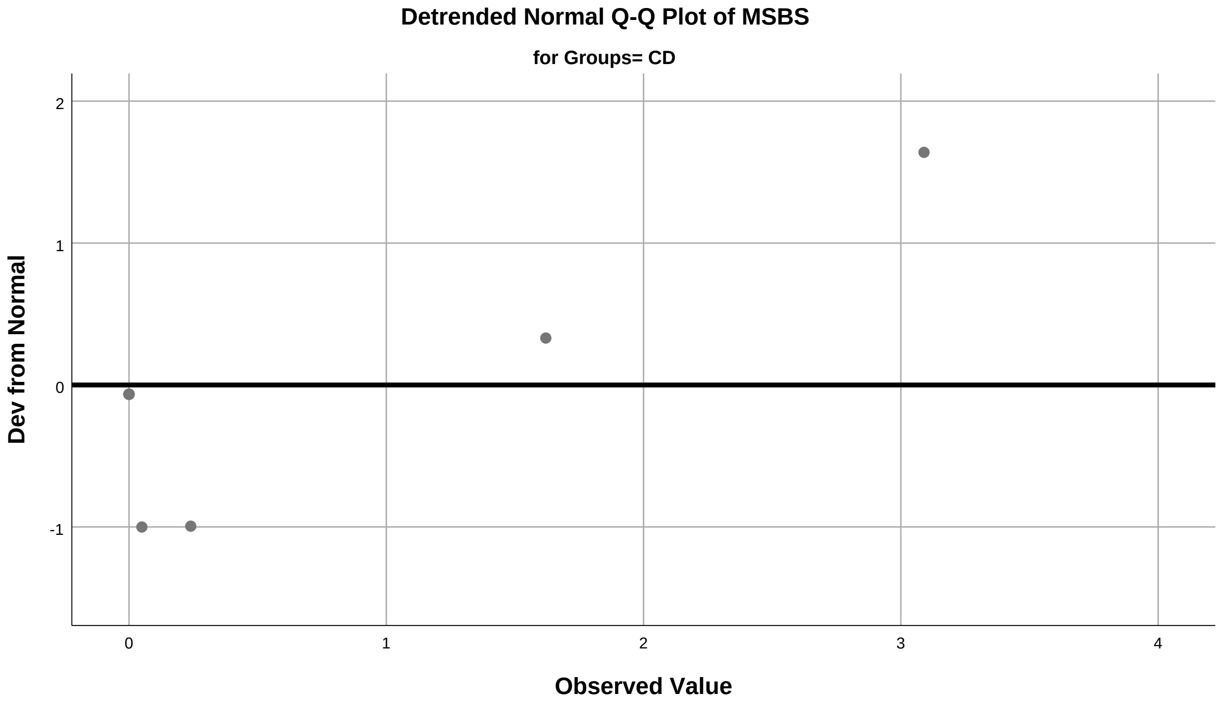
Detrended Normal Q-Q Plot of MSBS
for Groups= CD
Dev from Normal
2
1
0
-1
0
1
2
3
4
Observed Value

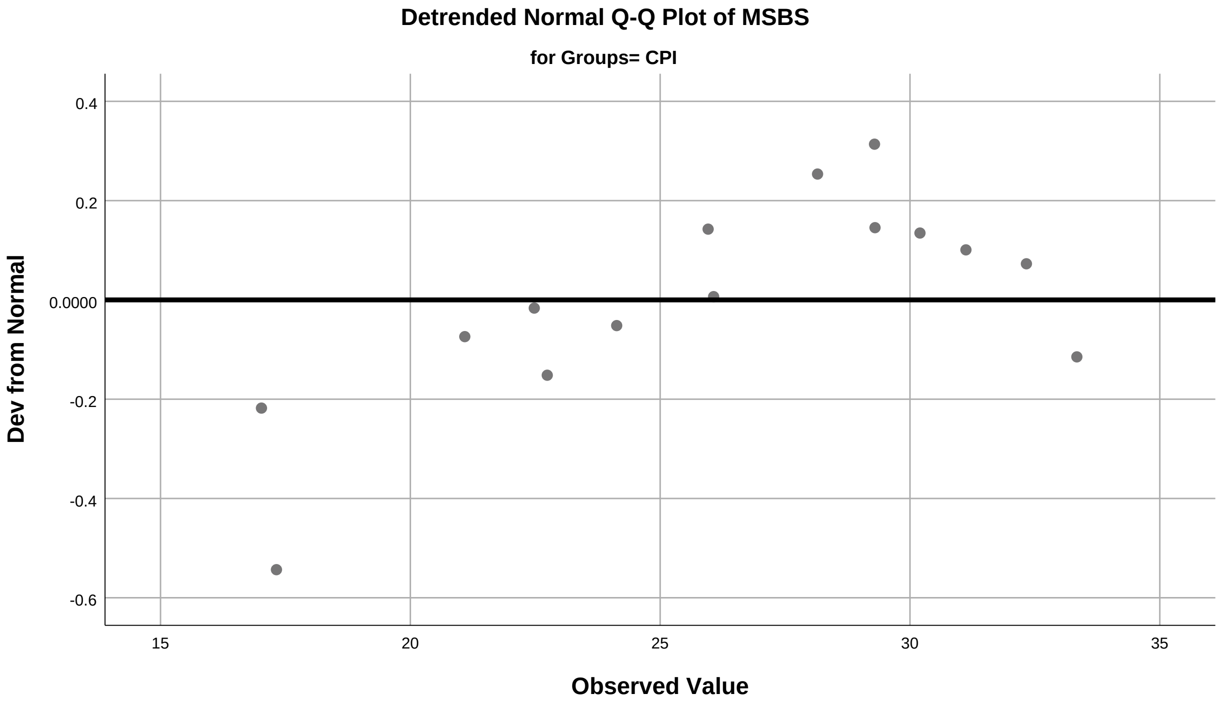
Detrended Normal Q-Q Plot of MSBS
for Groups= CPI
Dev from Normal
0.4
0.2
0.0000
-0.2
-0.4
-0.6
15
20
25
30
35
Observed Value

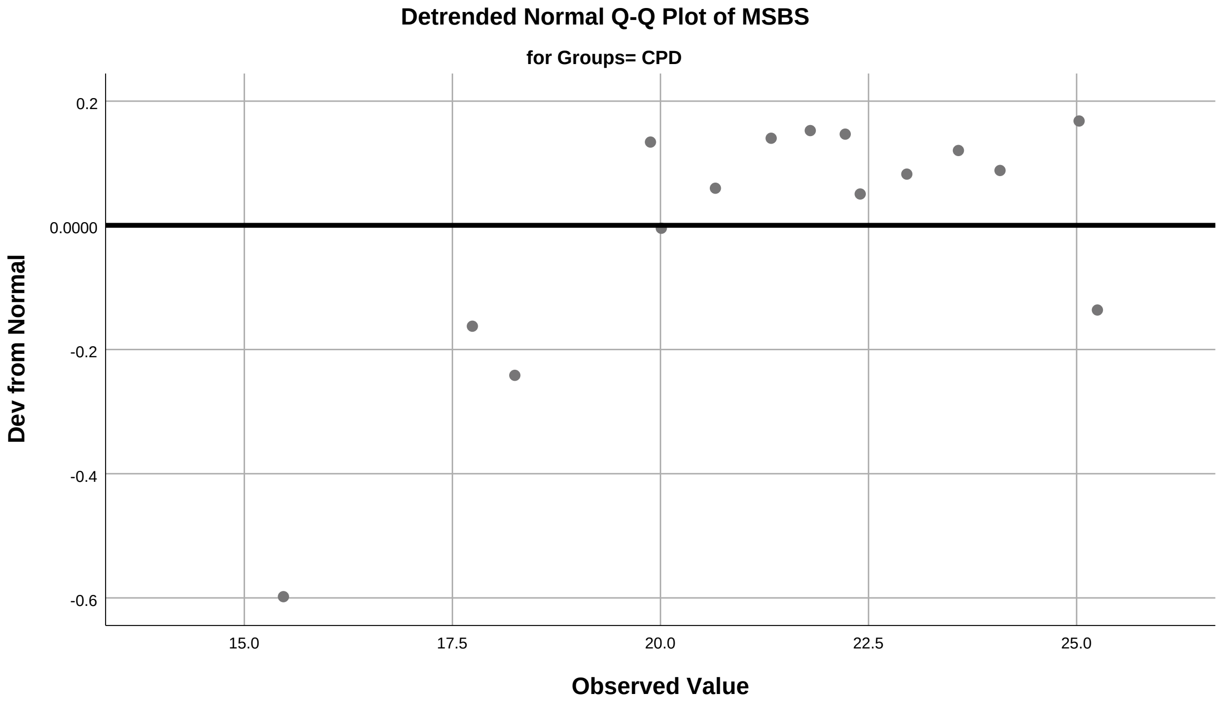
Detrended Normal Q-Q Plot of MSBS
for Groups= CPD
Dev from Normal
0.2
0.0000
-0.2
-0.4
-0.6
15.0
17.5
20.0
22.5
25.0
Observed Value

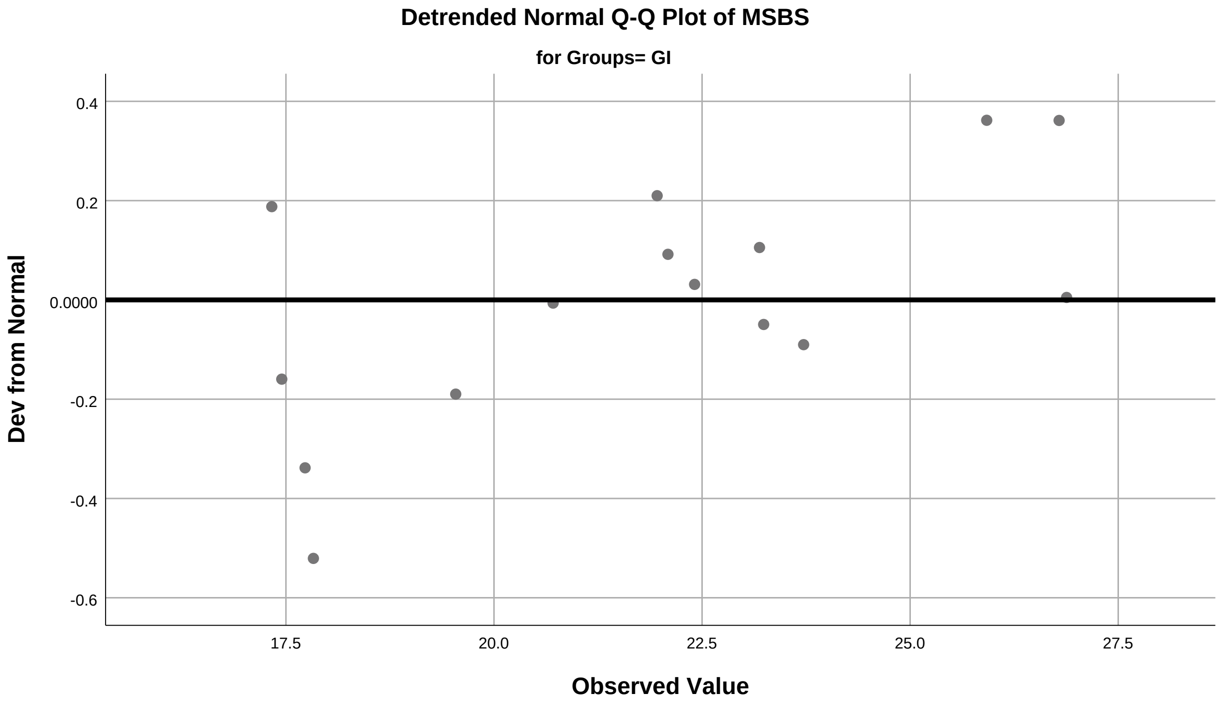

Detrended Normal Q-Q Plot of MSBS
for Groups= GI
Dev from Normal
0.4
0.2
0.0000
-0.2
-0.4
-0.6
17.5
20.0
22.5
25.0
27.5
Observed Value

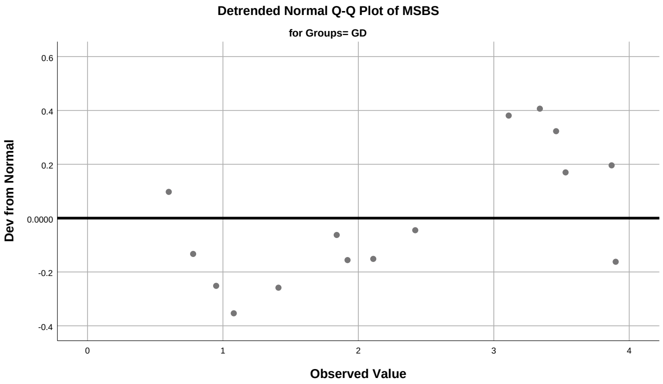

Detrended Normal Q-Q Plot of MSBS
for Groups= GD
Dev from Normal
0.6
0.4
0.2
0.0000
-0.2
-0.4
0
1
2
3
4
Observed Value

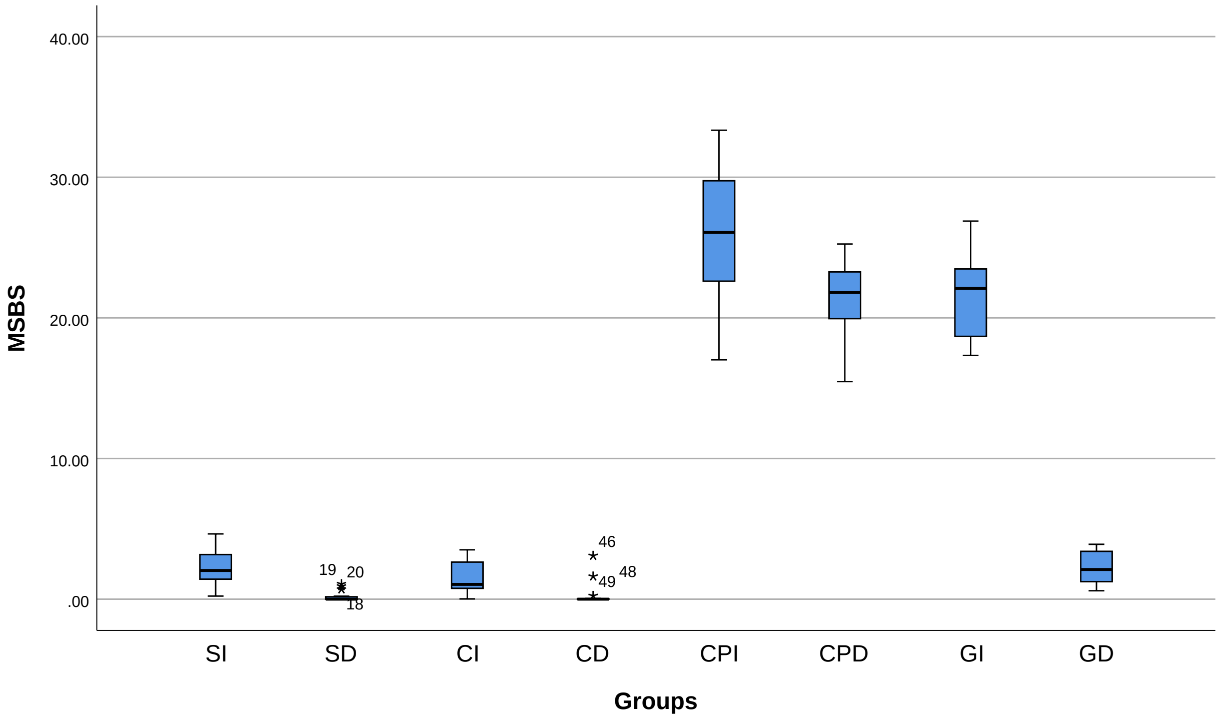


```
UNIANOVA MSBS BY Rest Time
 /METHOD=SSTYPE(3)
 /INTERCEPT=INCLUDE
 /CRITERIA=ALPHA(0.05)
 /DESIGN=Rest Time Rest*Time.
```

Univariate Analysis of Variance

Notes

Output Created		04-MAR-2024 10:07:50
Comments		
Input	Data	C:\Users\2 M TECH\Desktop\raw.sav1.sav
	Active Dataset	DataSet1
	Filter	<none>
	Weight	<none>
	Split File	<none>
	N of Rows in Working Data File	120
Missing Value Handling	Definition of Missing	User-defined missing values are treated as missing.
	Cases Used	Statistics are based on all cases with valid data for all variables in the model.
Syntax		UNIANOVA MSBS BY Rest Time /METHOD=SSTYPE(3) /INTERCEPT=INCLUDE /CRITERIA=ALPHA(0.05) /DESIGN=Rest Time ...
Resources	Processor Time	00:00:00.05
	Elapsed Time	00:00:00.09

Between-Subjects Factors

		Value Label	N
Rest	1	S	30
	2	C	30
	3	CP	30
	4	G	30
Time	1	I	60
	2	D	60

Tests of Between-Subjects Effects

Dependent Variable: MSBS

Source	Type III Sum of Squares	df	Mean Square	F	Sig.
Corrected Model	13573.513[a]	7	1939.073	309.351	.000
Intercept	10755.837	1	10755.837	1715.937	.000
Rest	10519.678	3	3506.559	559.420	.000
Time	1400.492	1	1400.492	223.428	.000
Rest * Time	1653.344	3	551.115	87.922	.000
Error	702.038	112	6.268		
Total	25031.389	120			
Corrected Total	14275.552	119			

a. R Squared = .951 (Adjusted R Squared = .948)

```
ONEWAY MSBS BY Groups
 /STATISTICS DESCRIPTIVES HOMOGENEITY
 /MISSING ANALYSIS
 /POSTHOC=TUKEY BONFERRONI ALPHA(0.05).
```

Oneway

Notes

Output Created		04-MAR-2024 10:08:55
Comments		
Input	Data	C:\Users\2 M TECH\Desktop\raw.sav1.sav
	Active Dataset	DataSet1
	Filter	<none>
	Weight	<none>
	Split File	<none>
	N of Rows in Working Data File	120
Missing Value Handling	Definition of Missing	User-defined missing values are treated as missing.
	Cases Used	Statistics for each analysis are based on cases with no missing data for any variable in the analysis.
Syntax		ONEWAY MSBS BY Groups /STATISTICS DESCRIPTIVES HOMOGENEITY /MISSING ANALYSIS /POSTHOC=TUKEY BONFERRONI ALPHA (0.05).
Resources	Processor Time	00:00:00.06
	Elapsed Time	00:00:00.11

Descriptives

MSBS

	N	Mean	Std. Deviation	Std. Error	95% Confidence Interval for Mean		Minimum
					Lower Bound	Upper Bound	
SI	15	2.1587	1.24608	.32174	1.4686	2.8487	.22
SD	15	.2060	.37127	.09586	.0004	.4116	.00
CI	15	1.5540	1.09871	.28368	.9456	2.1624	.02
CD	15	.3333	.86874	.22431	-.1478	.8144	.00
CPI	15	26.0360	5.14593	1.32867	23.1863	28.8857	17.02
CPD	15	21.3773	2.77064	.71538	19.8430	22.9117	15.47
GI	15	21.7860	3.30991	.85461	19.9530	23.6190	17.33
GD	15	2.2880	1.17500	.30338	1.6373	2.9387	.60
Total	120	9.4674	10.95274	.99984	7.4876	11.4472	.00

Descriptives

MSBS

	Maximum
SI	4.64
SD	1.08
CI	3.51
CD	3.09
CPI	33.34
CPD	25.25
GI	26.88
GD	3.90
Total	33.34

Test of Homogeneity of Variances

		Levene Statistic	df1	df2	Sig.
MSBS	Based on Mean	14.100	7	112	.000
	Based on Median	13.411	7	112	.000
	Based on Median and with adjusted df	13.411	7	47.203	.000
	Based on trimmed mean	14.276	7	112	.000

ANOVA

MSBS

	Sum of Squares	df	Mean Square	F	Sig.
Between Groups	13573.513	7	1939.073	309.351	.000
Within Groups	702.038	112	6.268		
Total	14275.552	119			

Post Hoc Tests

Multiple Comparisons

Dependent Variable: MSBS

	(I) Groups	(J) Groups	Mean Difference (I-J)	Std. Error	Sig.	95% ... Lower Bound
Tukey HSD	SI	SD	1.95267	.91420	.399	-.8713
		CI	.60467	.91420	.998	-2.2193
		CD	1.82533	.91420	.489	-.9987
		CPI	-23.87733*	.91420	.000	-26.7013
		CPD	-19.21867*	.91420	.000	-22.0427
		GI	-19.62733*	.91420	.000	-22.4513
		GD	-.12933	.91420	1.000	-2.9533
	SD	SI	-1.95267	.91420	.399	-4.7767
		CI	-1.34800	.91420	.819	-4.1720
		CD	-.12733	.91420	1.000	-2.9513
		CPI	-25.83000*	.91420	.000	-28.6540
		CPD	-21.17133*	.91420	.000	-23.9953
		GI	-21.58000*	.91420	.000	-24.4040
		GD	-2.08200	.91420	.315	-4.9060
	CI	SI	-.60467	.91420	.998	-3.4287
		SD	1.34800	.91420	.819	-1.4760
		CD	1.22067	.91420	.883	-1.6033
		CPI	-24.48200*	.91420	.000	-27.3060
		CPD	-19.82333*	.91420	.000	-22.6473
		GI	-20.23200*	.91420	.000	-23.0560
		GD	-.73400	.91420	.993	-3.5580
	CD	SI	-1.82533	.91420	.489	-4.6493
		SD	.12733	.91420	1.000	-2.6967

Multiple Comparisons

Dependent Variable: MSBS

	(I) Groups	(J) Groups	95% Confidence . Upper Bound
Tukey HSD	SI	SD	4.7767
		CI	3.4287
		CD	4.6493
		CPI	-21.0533
		CPD	-16.3947
		GI	-16.8033
		GD	2.6947
	SD	SI	.8713
		CI	1.4760
		CD	2.6967
		CPI	-23.0060
		CPD	-18.3473
		GI	-18.7560
		GD	.7420
	CI	SI	2.2193
		SD	4.1720
		CD	4.0447
		CPI	-21.6580
		CPD	-16.9993
		GI	-17.4080
		GD	2.0900
	CD	SI	.9987
		SD	2.9513

Multiple Comparisons

Dependent Variable: MSBS

(I) Groups	(J) Groups	Mean Difference (I-J)	Std. Error	Sig.	95% ... Lower Bound
	CI	-1.22067	.91420	.883	-4.0447
	CPI	-25.70267*	.91420	.000	-28.5267
	CPD	-21.04400*	.91420	.000	-23.8680
	GI	-21.45267*	.91420	.000	-24.2767
	GD	-1.95467	.91420	.397	-4.7787
CPI	SI	23.87733*	.91420	.000	21.0533
	SD	25.83000*	.91420	.000	23.0060
	CI	24.48200*	.91420	.000	21.6580
	CD	25.70267*	.91420	.000	22.8787
	CPD	4.65867*	.91420	.000	1.8347
	GI	4.25000*	.91420	.000	1.4260
	GD	23.74800*	.91420	.000	20.9240
CPD	SI	19.21867*	.91420	.000	16.3947
	SD	21.17133*	.91420	.000	18.3473
	CI	19.82333*	.91420	.000	16.9993
	CD	21.04400*	.91420	.000	18.2200
	CPI	-4.65867*	.91420	.000	-7.4827
	GI	-.40867	.91420	1.000	-3.2327
	GD	19.08933*	.91420	.000	16.2653
GI	SI	19.62733*	.91420	.000	16.8033
	SD	21.58000*	.91420	.000	18.7560
	CI	20.23200*	.91420	.000	17.4080
	CD	21.45267*	.91420	.000	18.6287
	CPI	-4.25000*	.91420	.000	-7.0740
	CPD	.40867	.91420	1.000	-2.4153
	GD	19.49800*	.91420	.000	16.6740
GD	SI	.12933	.91420	1.000	-2.6947
	SD	2.08200	.91420	.315	-.7420
	CI	.73400	.91420	.993	-2.0900
	CD	1.95467	.91420	.397	-.8693
	CPI	-23.74800*	.91420	.000	-26.5720

Multiple Comparisons

Dependent Variable: MSBS

	(I) Groups	(J) Groups	95% Confidence . Upper Bound
		CI	1.6033
		CPI	-22.8787
		CPD	-18.2200
		GI	-18.6287
		GD	.8693
	CPI	SI	26.7013
		SD	28.6540
		CI	27.3060
		CD	28.5267
		CPD	7.4827
		GI	7.0740
		GD	26.5720
	CPD	SI	22.0427
		SD	23.9953
		CI	22.6473
		CD	23.8680
		CPI	-1.8347
		GI	2.4153
		GD	21.9133
	GI	SI	22.4513
		SD	24.4040
		CI	23.0560
		CD	24.2767
		CPI	-1.4260
		CPD	3.2327
		GD	22.3220
	GD	SI	2.9533
		SD	4.9060
		CI	3.5580
		CD	4.7787
		CPI	-20.9240

Multiple Comparisons

Dependent Variable: MSBS

	(I) Groups	(J) Groups	Mean Difference (I-J)	Std. Error	Sig.	95% ... Lower Bound
		CPD	-19.08933*	.91420	.000	-21.9133
		GI	-19.49800*	.91420	.000	-22.3220
Bonferroni	SI	SD	1.95267	.91420	.976	-.9732
		CI	.60467	.91420	1.000	-2.3212
		CD	1.82533	.91420	1.000	-1.1005
		CPI	-23.87733*	.91420	.000	-26.8032
		CPD	-19.21867*	.91420	.000	-22.1445
		GI	-19.62733*	.91420	.000	-22.5532
		GD	-.12933	.91420	1.000	-3.0552
	SD	SI	-1.95267	.91420	.976	-4.8785
		CI	-1.34800	.91420	1.000	-4.2738
		CD	-.12733	.91420	1.000	-3.0532
		CPI	-25.83000*	.91420	.000	-28.7558
		CPD	-21.17133*	.91420	.000	-24.0972
		GI	-21.58000*	.91420	.000	-24.5058
		GD	-2.08200	.91420	.690	-5.0078
	CI	SI	-.60467	.91420	1.000	-3.5305
		SD	1.34800	.91420	1.000	-1.5778
		CD	1.22067	.91420	1.000	-1.7052
		CPI	-24.48200*	.91420	.000	-27.4078
		CPD	-19.82333*	.91420	.000	-22.7492
		GI	-20.23200*	.91420	.000	-23.1578
		GD	-.73400	.91420	1.000	-3.6598
	CD	SI	-1.82533	.91420	1.000	-4.7512
		SD	.12733	.91420	1.000	-2.7985
		CI	-1.22067	.91420	1.000	-4.1465
		CPI	-25.70267*	.91420	.000	-28.6285
		CPD	-21.04400*	.91420	.000	-23.9698
		GI	-21.45267*	.91420	.000	-24.3785
		GD	-1.95467	.91420	.971	-4.8805
	CPI	SI	23.87733*	.91420	.000	20.9515
		SD	25.83000*	.91420	.000	22.9042

Multiple Comparisons

Dependent Variable: MSBS

	(I) Groups	(J) Groups	95% Confidence . Upper Bound
		CPD	-16.2653
		GI	-16.6740
Bonferroni	SI	SD	4.8785
		CI	3.5305
		CD	4.7512
		CPI	-20.9515
		CPD	-16.2928
		GI	-16.7015
		GD	2.7965
	SD	SI	.9732
		CI	1.5778
		CD	2.7985
		CPI	-22.9042
		CPD	-18.2455
		GI	-18.6542
		GD	.8438
	CI	SI	2.3212
		SD	4.2738
		CD	4.1465
		CPI	-21.5562
		CPD	-16.8975
		GI	-17.3062
		GD	2.1918
	CD	SI	1.1005
		SD	3.0532
		CI	1.7052
		CPI	-22.7768
		CPD	-18.1182
		GI	-18.5268
		GD	.9712
	CPI	SI	26.8032
		SD	28.7558

Multiple Comparisons

Dependent Variable: MSBS

(I) Groups	(J) Groups	Mean Difference (I-J)	Std. Error	Sig.	95% ... Lower Bound
	CI	24.48200*	.91420	.000	21.5562
	CD	25.70267*	.91420	.000	22.7768
	CPD	4.65867*	.91420	.000	1.7328
	GI	4.25000*	.91420	.000	1.3242
	GD	23.74800*	.91420	.000	20.8222
CPD	SI	19.21867*	.91420	.000	16.2928
	SD	21.17133*	.91420	.000	18.2455
	CI	19.82333*	.91420	.000	16.8975
	CD	21.04400*	.91420	.000	18.1182
	CPI	-4.65867*	.91420	.000	-7.5845
	GI	-.40867	.91420	1.000	-3.3345
	GD	19.08933*	.91420	.000	16.1635
GI	SI	19.62733*	.91420	.000	16.7015
	SD	21.58000*	.91420	.000	18.6542
	CI	20.23200*	.91420	.000	17.3062
	CD	21.45267*	.91420	.000	18.5268
	CPI	-4.25000*	.91420	.000	-7.1758
	CPD	.40867	.91420	1.000	-2.5172
	GD	19.49800*	.91420	.000	16.5722
GD	SI	.12933	.91420	1.000	-2.7965
	SD	2.08200	.91420	.690	-.8438
	CI	.73400	.91420	1.000	-2.1918
	CD	1.95467	.91420	.971	-.9712
	CPI	-23.74800*	.91420	.000	-26.6738
	CPD	-19.08933*	.91420	.000	-22.0152
	GI	-19.49800*	.91420	.000	-22.4238

Multiple Comparisons

Dependent Variable: MSBS

(I) Groups	(J) Groups	95% Confidence . Upper Bound
	CI	27.4078
	CD	28.6285
	CPD	7.5845
	GI	7.1758
	GD	26.6738
CPD	SI	22.1445
	SD	24.0972
	CI	22.7492
	CD	23.9698
	CPI	-1.7328
	GI	2.5172
	GD	22.0152
GI	SI	22.5532
	SD	24.5058
	CI	23.1578
	CD	24.3785
	CPI	-1.3242
	CPD	3.3345
	GD	22.4238
GD	SI	3.0552
	SD	5.0078
	CI	3.6598
	CD	4.8805
	CPI	-20.8222
	CPD	-16.1635
	GI	-16.5722

*. The mean difference is significant at the 0.05 level.

Homogeneous Subsets

MSBS

	Groups	N	Subset for alpha = 0.05 1	2	3
Tukey HSD[a]	SD	15	.2060		
	CD	15	.3333		
	CI	15	1.5540		
	SI	15	2.1587		
	GD	15	2.2880		
	CPD	15		21.3773	
	GI	15		21.7860	
	CPI	15			26.0360
	Sig.		.315	1.000	1.000

Means for groups in homogeneous subsets are displayed.

a. Uses Harmonic Mean Sample Size = 15.000.

```
 DATASET ACTIVATE DataSet1.

SAVE OUTFILE='C:\Users\2 M TECH\Desktop\raw.sav1.sav'
  /COMPRESSED.

>Warning # 5334.  Command name: SAVE
>The SAVE command has succeeded.  However, due to contention for the specified
>file, the data have been saved to a file with a different name.
>Saved to C:\Users\2 M TECH\Desktop\raw.sav1_1.sav.
DATASET ACTIVATE DataSet1.

SAVE OUTFILE='C:\Users\2 M TECH\Desktop\raw.sav1_1.sav  results.sav'
  /COMPRESSED.
```
